# Characterizing Medicine Quality by Active Pharmaceutical Ingredient Levels: A Systematic Review and Meta-Analysis across Low- and Middle-Income Countries

**DOI:** 10.4269/ajtmh.21-1123

**Published:** 2022-06-15

**Authors:** Sachiko Ozawa, Hui-Han Chen, Yi-Fang (Ashley) Lee, Colleen R. Higgins, Tatenda T. Yemeke

**Affiliations:** ^1^ Division of Practice Advancement and Clinical Education, UNC Eshelman School of Pharmacy, University of North Carolina, Chapel Hill, North Carolina;; ^2^ Department of Maternal and Child Health, UNC Gillings School of Global Public Health, University of North Carolina, Chapel Hill, North Carolina

## Abstract

Substandard and falsified medicines are often reported jointly, making it difficult to recognize variations in medicine quality. This study characterized medicine quality based on active pharmaceutical ingredient (API) amounts reported among substandard and falsified essential medicines in low- and middle-income countries (LMICs). A systematic review and meta-analysis was conducted using PubMed, supplemented by results from a previous systematic review, and the Medicine Quality Scientific Literature Surveyor. Study quality was assessed using the Medicine Quality Assessment Reporting Guidelines (MEDQUARG). Random-effects models were used to estimate the prevalence of medicines with < 50% API. Among 95,520 medicine samples from 130 studies, 12.4% (95% confidence interval [CI]: 10.2–14.6%) of essential medicines tested in LMICs were considered substandard or falsified, having failed at least one type of quality analysis. We identified 99 studies that reported API content, where 1.8% (95% CI: 0.8–2.8%) of samples reported containing < 50% of stated API. Among all failed samples (*N* = 9,724), 25.9% (95% CI: 19.3–32.6%) reported having < 80% API. Nearly one in seven (13.8%, 95% CI: 9.0–18.6%) failed samples were likely to be falsified based on reported API amounts of < 50%, whereas the remaining six of seven samples were likely to be substandard. Furthermore, 12.5% (95% CI: 7.7–17.3%) of failed samples reported finding 0% API. Many studies did not present a breakdown of actual API amount of each tested sample. We offer suggested improved guidelines for reporting poor-quality medicines. Consistent data on substandard and falsified medicines and medicine-specific tailored interventions are needed to ensure medicine quality throughout the supply chain.

## INTRODUCTION

Poor-quality medicines pose a significant threat to patients and health systems globally because they may be ineffective, resulting in increased length of illness and the need for further treatment.^1^^–^^5^ In worse cases, poor-quality medicines can cause severe adverse reactions or lack life-saving active ingredients, resulting in avertable deaths.^2^^,^^3^^,^^6^^,^^7^ In 2017, the World Health Organization (WHO) adopted formal definitions of substandard and falsified medical products to describe poor-quality medicines.^8^ Substandard medicines refer to “authorized medical products that fail to meet either their quality standards or specifications, or both.”^6^ Falsified medicines are defined as “medical products that deliberately or fraudulently misrepresent their identity, composition, or source.”^6^

A variety of testing methods can detect substandard and falsified medicines, including visual and physical inspection, dissolution testing, and analysis of active pharmaceutical ingredient (API) content.^3^ A WHO review found that 1 in 10 essential medicines in low- and middle-income countries (LMICs) failed tests for quality.^2^ Two recent studies estimated a similar range from 13% to 25%.^9^^,^^10^ However, these analyses report all failed samples together, without distinguishing what pharmacopeia standards are being applied and how much the failed samples deviated from these specifictions.^11^ Understanding how much substandard and falsified medicines deviate from pharmacopeial standards for API content would add needed depth to the interpretation of overall prevalence of poor-quality medicines, and has implications for interventions to address them.^11^

Although many quality attributes (e.g., disintegration, dissolution, degradation, and presence of impurities) can affect treatment outcomes, the API content of a medicine is highly associated with its therapeutic efficacy and has implications for the development of antimicrobial resistance.^12^ Broadly, medicines with insufficient API content reduce therapeutic efficacy and have a more extensive impact on resistance compared with medicines with no API.^12^

The extent of deviation in the API content can indicate where supply chain issues are and allow for better tailoring of interventions. For example, some drugs may deviate only slightly from specifications, most likely indicating inadequate manufacturing or poor storage conditions. On the other hand, medicines with substantially low amounts of API, no API, or an incorrect API may indicate fraud, which may be further investigated by the pharmaceutical company or national medicines regulatory authorities (NMRAs). Because manufacturing falsified medicines is criminal, substandard and falsified medicines have different legal ramifications and require distinct solutions. A 2016 report on quality of lifesaving medicines differentiated samples by levels of deviation to understand the therapeutic effects of the products.^13^ However, studies differentiating poor-quality medicines by API content levels have not previously been documented.

This systematic review and meta-analysis updates prior analyses^9^ and seeks to break down the prevalence of substandard and falsified essential medicines in LMICs by API levels. We examined amounts of API content among essential medicines in studies that tested medicine quality in LMICs. We also offer guidance on how to improve reporting of poor-quality medicines in future medicine quality studies.

## MATERIALS AND METHODS

### Systematic review.

We searched for medicine quality studies in LMICs. First, we used searches from PubMed, EconLit, Global Health, Embase, and Scopus covering publications up to November 3, 2017.^9^ Search terms involved iterations of the terms “substandard and falsified medicines,” “quality of medicines,” and “low- and middle-income countries.” Second, we updated this search in PubMed to February 4, 2020. Third, we searched the Infectious Diseases Data Observatory’s Medicine Quality Scientific Literature Surveyor, an online platform that gathers medicine quality studies, from inception through September 10, 2020. This database reviews PubMed, Google Scholar, Embase, the WHO, the U.S. Pharmacopeia, Medical Regulatory Agencies’ websites, and other sources to include scientific reports on medicine quality in English, French, and Spanish.^14^ Further details of the search strategy and search terms are included in the supplemental materials.

Studies were included in the systematic review if they assessed medicine quality, examined essential medicines as classified by the WHO, were conducted in LMICs as classified by the World Bank, and reported the quantity of samples tested and failed.^15^ Included studies reported original sampling and testing data where samples were taken or purchased directly from markets. To ensure adequate statistical power and study quality, we included studies that tested a minimum of 50 samples. Studies without primary data, publications without full texts, and case reports were excluded.

After removing duplicates, each publication was independently reviewed for potential inclusion by two of four reviewers (H. C., Y. L., C. H., and T. Y.) based on the title and abstract, followed by a full-text review. Any inconsistencies between dual reviewers were addressed by a third independent reviewer (S.O.). Data abstraction was completed independently by three abstractors (H.C., Y.L., and C.H.). Discrepancies between abstracted results were discussed and resolved between the abstractors and S. O. Study data, including the sample size, type of sampling and testing methods, publication year, country where samples were collected, medicine class, and the number of samples tested and failed were extracted in Excel.

We used the 12-item Medicine Quality Assessment Reporting Guidelines (MEDQUARG) to evaluate the reporting standard of medicine quality studies.^16^^,^^17^ Studies not included in the previous review were rated by two reviewers (H. C., Y. L.). A Spearman’s correlation coefficient between reviewers was assessed for interrater reliability. Further information on MEDQUARG scoring and interrater reliability is reported in the supplemental materials.

### Meta-analysis across substandard and falsified samples.

Two separate meta-analyses were conducted. First, we estimated the prevalence of substandard and falsified medicines across all studies that assessed medicine quality in LMICs using a random-effects model, taking into account study sample sizes and MEDQUARG scores. A subgroup analysis was performed to illustrate the variation in the average weighted prevalence of substandard and falsified medicines across regions and therapeutic categories.

To assess the heterogeneity across studies, we evaluated the results of the random-effects model based on Cochran’s *Q* and *I*^2^. Effect modifiers were assessed to identify study features that may be associated with heterogeneity across studies included in the meta-analysis. We tested five potential effect modifiers using a mixed-effects model: publication year, region, medicine category, number of samples tested, and MEDQUARG scores. A Baujat plot analysis was conducted to examine the influence of each study on pooled results.^18^ A funnel plot and funnel plot asymmetry test assessed potential publication bias.^19^ Additionally, we examined which studies exerted the most influence on the pooled weighted result using an influence plot analysis.^20^ These results are reported in the supplemental materials.

### Meta-analysis among samples that reported API levels.

A second meta-analysis was conducted among studies that reported API amounts in medicine samples tested. Studies were included if they reported the percentage API of all failed samples or reported the number of samples within API ranges. Studies that reported adequate data were included whether they found any substandard or falsified medicines.

We recorded the number of failed medicine samples reported into categories of API level deviations. We documented whether failed samples were reported to contain 1) no and/or incorrect API, 2) < 50% API, or 3) < 80% API. These categories were not mutually exclusive where samples could be classified into more than one category. For example, a sample with 0% API was included in counts containing < 50% API and in the classification for < 80% API. On the other hand, a sample that reported to have < 80% API without specifying the actual API amount was only included in the < 80% API category. Medicine samples with < 80% of API are considered to be “extremely deviating” from specifications and in the absence of evidence of falsification these medicines can be considered likely substandard,^13^ whereas those with < 50% of API can be considered likely falsified.^11^ Where available, we categorized samples that were documented as having incorrect labeling or false packaging because this is a common sign of falsification. We also recorded when authors claimed the samples were falsified without presenting data.

We estimated the pooled prevalence of medicines with 0% API and/or incorrect API, medicines with < 50% API, and medicines with < 80% API using random-effects models weighted by sample size and MEDQUARG scores. Studies with larger samples and higher MEDQUARG scores contributed greater weight. A subgroup analysis was conducted to examine the variation in API levels across regions and therapeutic categories.

This systematic review and meta-analysis was registered in the international prospective register of systematic reviews (PROSPERO) database (#CRD42020188678). Results are reported in line with the Preferred Reporting Items for Systematic Reviews and Meta-analyses (PRISMA) guidelines.

## RESULTS

### Systematic review.

**Figure 1.**
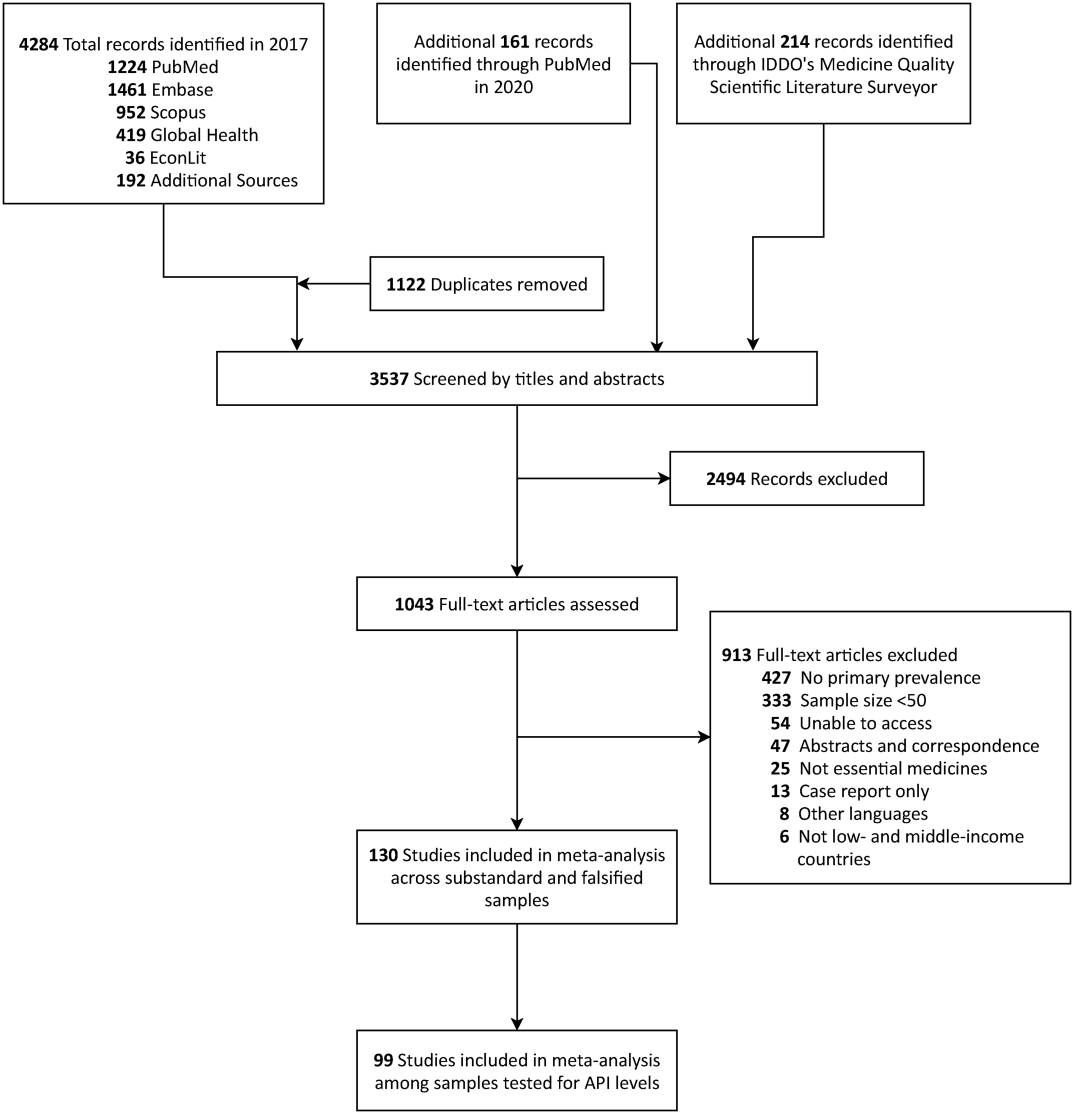
Preferred Reporting Items for Systematic Reviews and Meta-analyses (PRISMA) diagram.

Combined searches resulted in a total of 3,537 articles after removing duplicates, which were screened based on titles and abstracts. After conducting full-text screening of 1,043 studies, 130 studies were included in this systematic review (Figure 1; see supplemental materials for a list of studies).

Africa (58 studies, 44.6%) and Asia (48 studies, 36.9%) were the primary regions where medicine quality studies were conducted in LMICs, with few studies in South America (*N* = 5, 3.8%),^21^^–^^25^ Europe (*N* = 1, 0.8%),^26^ and Oceania (*N* = 1, 0.8%).^27^ In addition, we identified 17 studies (13.1%) that collected samples from multiple regions. The majority of the included articles (87 studies, 66.9%) were published in or after 2010, of which 31 studies (23.8%) were published since 2017. Antibiotics (74 studies) and antimalarials (70 studies) remain the most examined therapeutic classes for medicine quality. Additional classes of medicines that were tested for quality and reported in LMICs included analgesics and anti-inflammatories (27 studies), antihypertensives (17 studies), uterotonics (10 studies), steroids (10 studies), antidiabetics (8 studies), antiparasitics (8 studies), antiretrovirals (8 studies), and others such as vitamins, anticonvulsants, proton pump inhibitors, bronchodilators, opioids, and antifungals (24 studies). Across 130 studies, 95,520 samples were tested in total, with a median sample size of 248 samples per study and an interquartile range of 107 to 544 samples.

### Meta-analysis across substandard and falsified samples.

**Figure 2.**
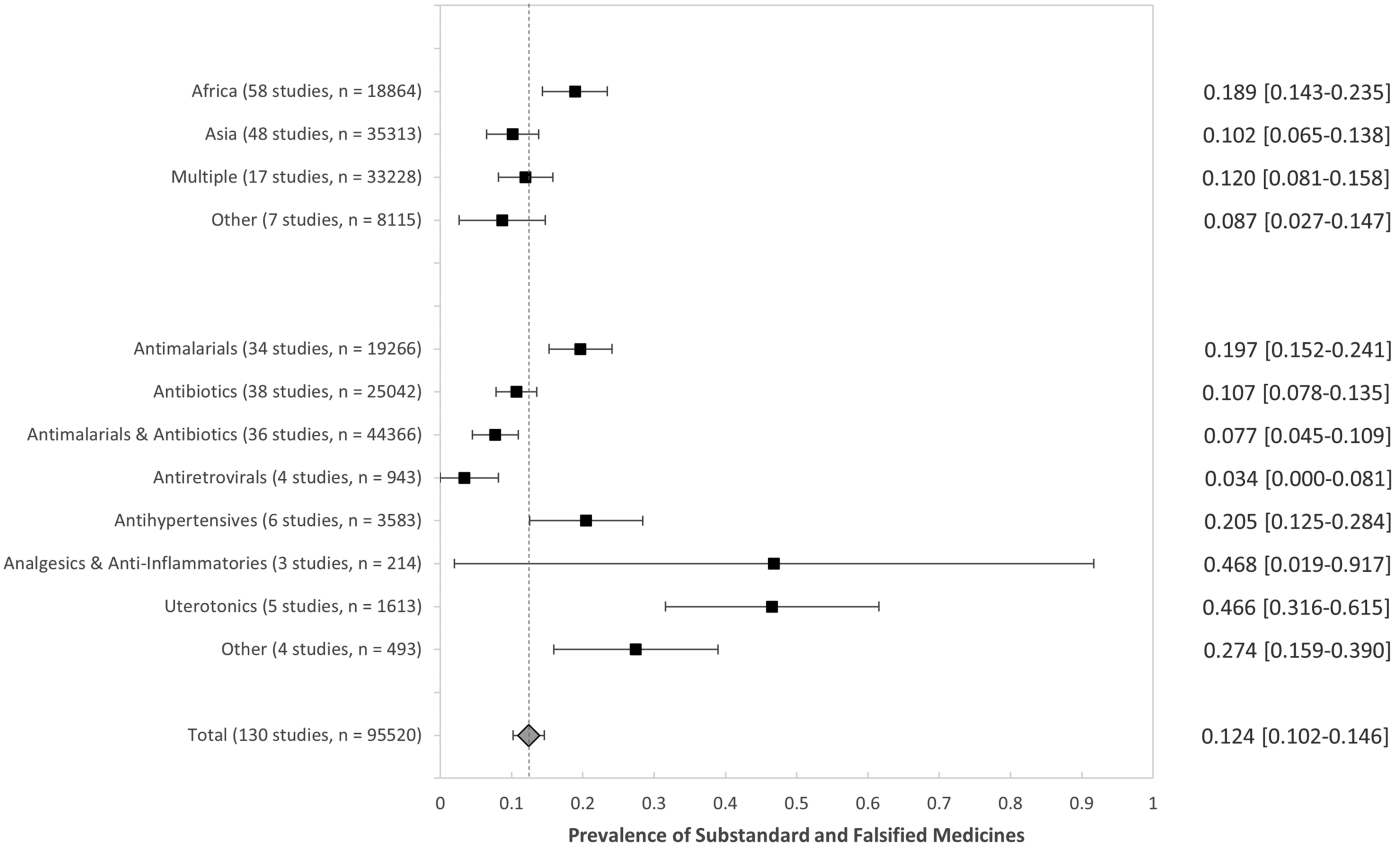
Forest plot of overall prevalence of substandard and falsified medicines. Sample size includes all medicine quality study samples tested. Antimalarials include studies that examined antimalarials but not antibiotics. Antibiotics exclude studies that examined antimalarials. Antimalarials and antibiotics category includes studies that examined both together. Sample sizes of 1) antiretrovirals, 2) antihypertensives, 3) analgesics and anti-inflammatories, and 4) uterotonics include studies that investigated the specific therapeutic category but not antibiotics or antimalarials, and may or may not include other therapeutic categories.

Figure 2 presents a forest plot of the weighted prevalence of substandard and falsified essential medicines across 130 included studies, with subgroup analyses by region and medication category. The overall weighted prevalence of substandard and falsified medicines in LMICs was 12.4% (95% CI: 10.2–14.6%) across all therapeutic categories and geographic regions. Substandard and falsified medicines were most prevalent in Africa at 18.9% (95% CI: 14.3– 23.5%), followed by Asia at 10.2% (95% CI: 6.5–13.8%), and in other single-region studies at 8.7% (95% CI: 2.7–14.7%). Among studies that combined samples from multiple regions, the prevalence of substandard and falsified medicines was estimated at 12.0% (95% CI: 8.1–15.8%).

Across 27 studies (*N* = 10,719) that examined labeling, 2.5% (95% CI: 0.5–4.4%) of labels were incorrect. Among six studies (*N* = 11,024) that did not offer data on samples,^28^^–^^33^ 1.5% (95% CI: 0.6–2.3%) were samples authors claimed were falsified.

Across therapeutic classes, substandard and falsified medicines were most prevalent among analgesics and antiinflammatories at 46.8% (95% CI: 1.9–91.7%), and uterotonics at 46.6% (95% CI: 31.6–61.5%), although both demonstrated large uncertainty due to small numbers of studies and samples tested. The prevalence of substandard and falsified antihypertensives was 20.5% (95% CI: 12.5–28.4%), antimalarials was 19.7% (95% CI: 15.2–24.1%), and antibiotics was 10.7% (95% CI: 7.8–13.5%). Among studies that combined the results of antimalarials and antibiotics, 7.7% (95% CI: 4.5–10.9%) of medicines were found to be substandard or falsified. Antiretrovirals were found to have the lowest substandard and falsified prevalence among therapeutic classes of medicines tested at 3.4% (95% CI: 0.0–8.1%).

The random-effects model showed considerable heterogeneity between studies (*I*^2^ = 99.92%) and funnel plot asymmetry showed publication bias (*P* < 0.001). Effect modifiers for the number of samples tested and region were significant (*P* < 0.05) in explaining some of the heterogeneity between studies (see supplemental materials). The heterogeneity demonstrated that studies in Africa and Asia, and those testing fewer samples tended to have higher prevalence of substandard and falsified medicines.

### Meta-analysis among samples that reported API levels.

From the second meta-analysis, we found that 99 of the 130 studies (76.2%) included information on API levels (Table 1).^4^^,^^13^^,^^21^^–^^24^^,^^26^^–^^31^^,^^34^^–^^120^ Many studies reported the number of samples that [Bibr b71][Bibr b72][Bibr b73][Bibr b74][Bibr b75][Bibr b76][Bibr b77][Bibr b78][Bibr b79][Bibr b80][Bibr b81][Bibr b82][Bibr b83][Bibr b84][Bibr b85][Bibr b86][Bibr b87][Bibr b88][Bibr b89][Bibr b90][Bibr b91][Bibr b92][Bibr b93][Bibr b94][Bibr b95][Bibr b96][Bibr b97][Bibr b98][Bibr b99][Bibr b100][Bibr b101][Bibr b102][Bibr b103][Bibr b104][Bibr b105][Bibr b106][Bibr b107][Bibr b108][Bibr b109][Bibr b110][Bibr b111][Bibr b112][Bibr b113][Bibr b114][Bibr b115][Bibr b116][Bibr b117][Bibr b118][Bibr b119]contained API amounts below a cutoff rather than the exact API amount of each sample. Overall weighted prevalence of medicines that were reported to contain < 50% API was 1.8% (95% CI: 0.8–2.8%) across all essential medicines in LMICs (see supplemental materials for forest plot). Regional prevalence of medicines with < 50% API was marginally higher in Asia and Africa at 2.8% (95% CI: 0.0–5.6%) and 2.2% (95% CI: 0.5–3.9%), respectively. On average, prevalence of medicines with < 50% API was 3.7% (95% CI: 0.0–8.8%) for uterotonics, 3.6% (95% CI: 0.1–7.2%) for antimalarials, and 1.6% (95% CI: 1.0–2.1%) for antibiotics. Across the 99 studies, we found that 1.6% (95% CI: 0.6–2.6%) of samples were reported to contain 0% API.

**Table 1:** Studies reporting active pharmaceutical ingredient (API) amounts of samples that failed medicine quality tests, by therapeutic class

Author (year)	Countries	Sample size	Incorrect or no API count (%)	< 50% API count (%)	< 80% API count (%)
**Analgesics and anti-inflammatories**					
Roy et al.^34^ (1993)	Bangladesh	53	0 (0.00%)	0 (0.00%)	16 (30.19%)
**Antibiotics**					
Alotaibi et al.^35^ (2018)	Haiti, Ghana, Sierra, Leone, Democratic Republic of Congo, India, Papua New Guinea, Ethiopia	290	0 (0.00%)	0 (0.00%)	4 (1.38%)
Bate et al.^36^ (2012)	Angola, Brazil, China, DRC, Egypt, Ethiopia, Ghana, India, Kenya, Mozambique, Nigeria, Russia, Rwanda, Tanzania, Thailand, Turkey, Uganda, Zambia	1,437	59 (4.11%)	59 (4.11%)	142 (9.88%)
Bate et al.^29^ (2013)	Angola, DRC, Egypt, Ethiopia, Ghana, Kenya, Nigeria, Rwanda, Tanzania, Uganda, Zambia, India, Thailand, China, Turkey, Russia, Brazil	713	0 (0.00%)	29 (4.07%)	65 (9.12%)
Bate et al.^37^ (2014)	Angola, DRC, Egypt, Ethiopia, Ghana, Kenya, Nigeria, Rwanda, Tanzania, Uganda, Zambia, India, Thailand, China, Turkey, Russia, Brazil, Mozambique	1,470	57 (3.88%)	57 (3.88%)	160 (10.88%)
Bate et al.^24^ (2018)	Argentina	687	14 (2.04%)	14 (2.04%)	48 (6.99%)
Boadu et al.^38^ (2015)	Ghana	54	0 (0.00%)	8 (14.81%)	16 (29.63%)
Exebio et al.^23^ (2010)	Peru	4,917	68 (1.38%)	68 (1.38%)	68 (1.38%)
Islam et al.^39^ (2018)	Myanmar	235	3 (1.28%)	3 (1.28%)	3 (1.28%)
Kamau et al.^40^ (2003)	Kenya	57	0 (0.00%)	2 (3.51%)	5 (8.77%)
Khan et al.^41^ (2013)	India	59	0 (0.00%)	0 (0.00%)	0 (0.00%)
Khurelbat et al.^42^ (2014)	Mongolia	1,236	0 (0.00%)	0 (0.00%)	0 (0.00%)
Khurelbat et al.^43^ (2020)	Mongolia	1,770	0 (0.00%)	0 (0.00%)	73 (4.12%)
Kumar et al.^44^ (2018)	India	3,925	90 (2.29%)	90 (2.29%)	110 (2.80%)
Kitutu et al.^45^ (2015)	Uganda	179	3 (1.68%)	3 (1.68%)	10 (5.59%)
Laserson et al.^46^ (2001)	Colombia, Estonia, India, Latvia, Russia, Vietnam	71	0 (0.00%)	0 (0.00%)	2 (2.82%)
Lawal et al.^47^ (2019)	Nigeria	112	3 (2.68%)	3 (2.68%)	39 (34.82%)
Myers et al.^48^ (2019)	Kenya	189	0 (0.00%)	0 (0.00%)	13 (6.88%)
Nabirova et al.^49^ (2017)	Kazakhstan	854	0 (0.00%)	0 (0.00%)	36 (4.22%)
Nazerali et al.^50^ (1998)	Zimbabwe	840	0 (0.00%)	0 (0.00%)	94 (11.19%)
Obaid et al.^51^ (2009)	Pakistan	96	0 (0.00%)	0 (0.00%)	3 (3.13%)
Patel et al.^52^ (2012)	South Africa	135	0 (0.00%)	0 (0.00%)	0 (0.00%)
Sabartova et al.^26^ (2011)	Armenia, Azerbaijan, Belarus, Estonia, Kazakhstan, Latvia, Moldova, Ukraine, Uzbekistan	291	0 (0.00%)	0 (0.00%)	1 (0.34%)
Sakolkhai et al.^53^ (1991)	Thailand	62	0 (0.00%)	0 (0.00%)	3 (4.84%)
Schafermann et al.^54^ (2018)	Togo	92	0 (0.00%)	1 (1.09%)	1 (1.09%)
Tabernero et al.^55^ (2019)	Laos	1,025	0 (0.00%)	0 (0.00%)	2 (0.20%)
Tshilumba et al.^56^ (2015)	Democratic Republic of Congo	60	0 (0.00%)	0 (0.00%)	0 (0.00%)
Wahidullah et al.^57^ (2011)	Afghanistan	348	0 (0.00%)	1 (0.29%)	1 (0.29%)
Wang et al.^58^ (2015)	South Africa, United States, China, Ethiopia, Thailand, Laos, Mexico, Nigeria	88	0 (0.00%)	0 (0.00%)	0 (0.00%)
WHO^13^ (2016)	Burkina Faso, Kenya, Madagascar, Nepal, Nigeria, Tajikistan, Tanzania, Uganda, Viet Nam, Zimbabwe	204	1 (0.49%)	1 (0.49%)	5 (2.45%)
Yoshida et al.^28^ (2014)	Cambodia	325	0 (0.00%)	0 (0.00%)	0 (0.00%)
**Antihypertensives**					
Antignac et al.^59^ (2017)	Benin, Burkina Faso, Republic of the Congo, the Democratic Republic of Congo, Guinea, Côte d’Ivoire, Mauritania, Niger, Senegal, Togo	1,530	0 (0.00%)	0 (0.00%)	24 (1.57%)
Ndichu et al.^60^ (2019)	Nigeria	102	0 (0.00%)	0 (0.00%)	6 (5.88%)
Rahman et al.^61^ (2019)	Cambodia	372	0 (0.00%)	6 (1.61%)	7 (1.88%)
Redfern et al.^62^ (2019)	Nigeria	361	0 (0.00%)	0 (0.00%)	0 (0.00%)
**Antimalarials**					
Amin et al.^63^ (2005)	Kenya	116	1 (0.86%)	1 (0.86%)	1 (0.86%)
Basco et al.^64^ (2004)	Cameroon	284	76 (26.76%)	76 (26.76%)	84 (29.58%)
Belew et al.^65^ (2019)	Ethiopia	74	0 (0.00%)	0 (0.00%)	0 (0.00%)
Bjorkman et al.^66^ (2012)	Uganda	558	108 (19.35%)	108 (19.35%)	108 (19.35%)
Dondorp et al.^67^ (2004)	Myanmar, Lao PDR, Vietnam, Cambodia, Thailand	232	99 (42.67%)	103 (44.40%)	103 (44.40%)
Evans et al.^21^ (2012)	Guyana and Suriname	135	2 (1.48%)	2 (1.48%)	12 (8.89%)
Guo et al.^68^ (2017)	Myanmar	153	1 (0.65%)	1 (0.65%)	1 (0.65%)
Idowu et al.^69^ (2006)	Nigeria	50	3 (6.00%)	3 (6.00%)	3 (6.00%)
Ioset et al.^70^ (2009)	13 countries in Asia, South America and Africa including Kenya, Nigeria, Vietnam; does not name all 13	171	2 (1.17%)	2 (1.17%)	2 (1.17%)
Kaur et al.^71^ (2008)	Tanzania	304	0 (0.00%)	0 (0.00%)	0 (0.00%)
Kaur et al.^72^ (2016)	Equatorial Guinea (Bioko Island), Cambodia, Ghana, Nigeria, Rwanda, Tanzania	10,079	98 (0.97%)	98 (0.97%)	98 (0.97%)
Khin et al.^73^ (2016)	Myanmar	51	2 (3.92%)	2 (3.92%)	2 (3.92%)
Lalani et al.^74^ (2015)	Afghanistan	134	0 (0.00%)	0 (0.00%)	0 (0.00%)
Maponga et al.^75^ (2003)	Gabon, Ghana, Kenya, Mali, Mozambique, Sudan, Zimbabwe	288	0 (0.00%)	0 (0.00%)	13 (4.51%)
Mufusama et al.^76^ (2018)	Democratic Republic of the Congo	150	4 (2.67%)	6 (4.00%)	19 (12.67%)
Mziray et al.^77^ (2017)	Tanzania	1,444	1 (0.07%)	1 (0.07%)	1 (0.07%)
Newton et al.^78^ (2001)	Cambodia, Laos, Myanmar, Thailand, Vietnam	104	39 (37.50%)	39 (37.50%)	39 (37.50%)
Newton et al.^79^ (2008)	Vietnam, Cambodia, Lao PDR, Myanmar, Thai/Myanmar border	391	195 (49.87%)	195 (49.87%)	195 (49.87%)
Ochekpe et al.^80^ (2010)	Nigeria	70	2 (2.86%)	2 (2.86%)	20 (28.57%)
Ogwal-Okeng et al.^81^ (1998)	Uganda	88	0 (0.00%)	0 (0.00%)	11 (12.50%)
Osei-Safo et al.^82^ (2014)	Ghana, Togo	124	1 (0.81%)	1 (0.81%)	6 (4.84%)
Phanouvong et al.^83^ (2013)	Cambodia	374	8 (2.14%)	17 (4.55%)	31 (8.29%)
Tabernero et al.^84^ (2015)	Laos	158	0 (0.00%)	0 (0.00%)	3 (1.90%)
Tipke et al.^85^ (2008)	Burkina Faso	77	1 (1.30%)	1 (1.30%)	13 (16.88%)
Visser et al.^86^ (2015)	Gabon	432	1 (0.23%)	2 (0.46%)	2 (0.46%)
WHO^87^ (2009)	Madagascar, Senegal, Uganda	197	0 (0.00%)	0 (0.00%)	0 (0.00%)
WHO^88^ (2011)	Cameroon, Ethiopia, Ghana, Kenya, Nigeria, Tanzania	267	2 (0.75%)	3 (1.12%)	8 (3.00%)
Yeung et al.^89^ (2015)	Cambodia	291	0 (0.00%)	2 (0.69%)	50 (17.18%)
**Antimalarials and antibiotics**					
Baratta et al.^90^ (2012)	Congo, Ethiopia, India, Malawi, CAR, Guinea Conakry, Uganda, Brazil, Guinea Bissau, Madagascar, Kenya, Angola, Rwanda, Cameroon, Chad	221	4 (1.81%)	4 (1.81%)	4 (1.81%)
Bate et al.^91^ (2010)	Ghana, Tanzania, Uganda, Nigeria, Angola, Zambia, Kenya, India, Thailand, China, Turkey, Russia, Brazil	2,065	0 (0.00%)	0 (0.00%)	210 (10.17%)
Central Drug Standard Control Organization^92^ (2009)	India	2,976	0 (0.00%)	0 (0.00%)	0 (0.00%)
Food and Drug Department^93^ (2010)	Lao	1,567	10 (0.64%)	10 (0.64%)	18 (1.15%)
Food and Drug Department^94^ (2014)	Lao	114	0 (0.00%)	0 (0.00%)	0 (0.00%)
Frimpong et al.^95^ (2018)	Ghana	68	0 (0.00%)	5 (7.35%)	15 (22.06%)
Hajjou et al.^96^ (2015)	Ghana, Ethiopia, Liberia, Kenya, and Mozambique, Cambodia, Indonesia, Laos, Myanmar, Philippines, Thailand, Vietnam, China, Colombia, Ecuador, Guyana, Peru	15,063	81 (0.54%)	81 (0.54%)	81 (0.54%)
Hetzel et al.^27^ (2014)	Papua New Guinea	360	0 (0.00%)	2 (0.56%)	25 (6.94%)
Kaale et al.^97^ (2016)	Tanzania	242	0 (0.00%)	5 (2.07%)	14 (5.79%)
Khan et al.^30^ (2011)	Cambodia	679	0 (0.00%)	0 (0.00%)	0 (0.00%)
Khuluza et al.^98^ (2017)	Malawi	56	1 (1.79%)	2 (3.57%)	3 (5.36%)
Kibwage et al.^99^ (1999)	Kenya	262	1 (0.38%)	1 (0.38%)	17 (6.49%)
Lon et al.^100^ (2006)	Cambodia	451	90 (19.96%)	90 (19.96%)	114 (25.28%)
Petersen et al.^4^ (2017)	Cameroon, Democratic Republic of the Congo, India, Ghana, Kenya, Nigeria, Uganda	869	12 (1.38%)	19 (2.19%)	20 (2.30%)
Phanouvong et al.^101^ (2013)	Thailand	709	4 (0.56%)	6 (0.85%)	6 (0.85%)
Pribluda et al.^22^ (2012)	Bolivia, Brazil, Colombia, Ecuador, Guyana, Suriname, Venezuela	1,663	0 (0.00%)	0 (0.00%)	0 (0.00%)
Risha et al.^31^ (2008)	Tanzania	1,257	0 (0.00%)	0 (0.00%)	0 (0.00%)
Schiavetti et al.^102^ (2018)	Democratic Republic of the Congo	239	0 (0.00%)	0 (0.00%)	8 (3.35%)
Seear et al.^103^ (2011)	India	300	0 (0.00%)	0 (0.00%)	0 (0.00%)
Shakoor et al.^104^ (1997)	Nigeria, Thailand	96	6 (6.25%)	6 (6.25%)	6 (6.25%)
Stenson et al.^105^ (1998)	Laos	366	12 (3.28%)	12 (3.28%)	17 (4.64%)
Syhakhang et al.^106^ (2002)	Laos	666	15 (2.25%)	15 (2.25%)	20 (3.00%)
Taylor et al.^107^ (2001)	Nigeria	581	6 (1.03%)	13 (2.24%)	32 (5.51%)
Uganda Medicines Transparency Alliance^108^ (2014)	Uganda	105	0 (0.00%)	0 (0.00%)	5 (4.76%)
Wondemagegnehu et al.^109^ (1999)	Myanmar, Vietnam	500	1 (0.20%)	3 (0.60%)	14 (2.80%)
WHO^110^ ( 1995)	Cameroon, Madagascar, Chad	429	17 (3.96%)	17 (3.96%)	58 (13.52%)
**Antiretrovirals**					
Kuwana et al.^111^ (2017)	Burkina Faso, Democratic Republic of the Congo, Nigeria, Rwanda, Zambia	126	0 (0.00%)	0 (0.00%)	0 (0.00%)
Ministry of Medical Services^112^ (2012)	Kenya	272	0 (0.00%)	0 (0.00%)	0 (0.00%)
WHO^113^ (2007)	Cameroon, Democratic Republic of the Congo, Kenya, Nigeria, Tanzania, Uganda, Zambia	394	0 (0.00%)	0 (0.00%)	0 (0.00%)
**Uterotonics**					
Anyakora et al.^114^ (2018)	Nigeria	637	0 (0.00%)	0 (0.00%)	0 (0.00%)
Hall et al.^115^ (2016)	Bangladesh, Egypt, Cambodia, Kenya, India, Mexico, Nigeria, Pakistan, Peru, Vietnam, Nigeria, Nepal, Pakistan, Bangladesh, Argentina, Indonesia, Peru, Philippines, Kazakhstan	215	14 (6.51%)	14 (6.51%)	14 (6.51%)
Karikari-Boateng et al.^116^ (2013)	Ghana	279	5 (1.79%)	5 (1.79%)	5 (1.79%)
Stanton et al.^117^ (2012)	Ghana	101	1 (0.99%)	25 (24.77%)	57 (56.40%)
Stanton et al.^118^ (2014)	India	381	0 (0.00%)	16 (4.20%)	44 (11.53%)
Other*					
Laroche et al.^119^ (2005)	Mauritania	146	0 (0.00%)	0 (0.00%)	5 (3.42%)
Suleman et al.^120^ (2014)	Ethiopia	106	0 (0.00%)	0 (0.00%)	1 (0.94%)

* Other includes phenobarbital, mebendazole, albendazole, and tinidazole.

**Figure 3.**
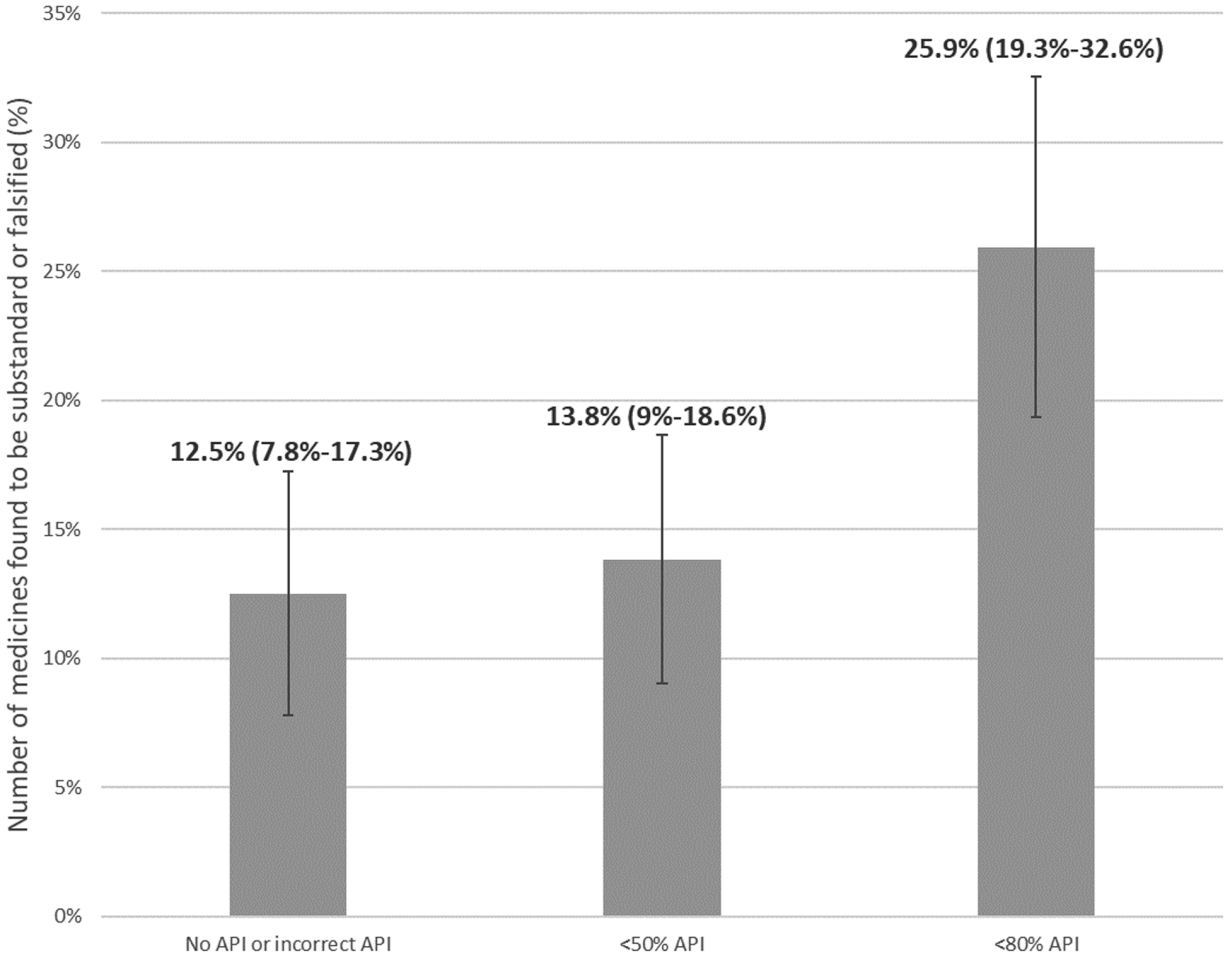
Proportion of samples that failed medicine quality tests by active pharmaceutical ingredient (API) levels. Sample size (99 studies, *N* = 9,724) includes studies with enough information to distinguish proportions of failed samples for no or incorrect API, > 50% API, and > 80% API.

Among the 99 studies that included information on API levels and found medication samples that failed quality testing (9,724 samples), we found 25.9% (95% CI: 19.3–32.6%) that were reported to contain < 80% API (Figure 3). The remainder failed other quality tests (e.g., disintegration, dissolution, degradation, presence of impurities, visual and physical inspection), contained API levels > 80% but below pharmacopeia standards or had API levels > 100%. Moreover, 13.8% (95% CI: 9.0–18.6%) of failed samples were reported to contain < 50% API, and 12.5% (95% CI: 7.8–17.3%) reported finding no or incorrect API.

**Figure 4.**
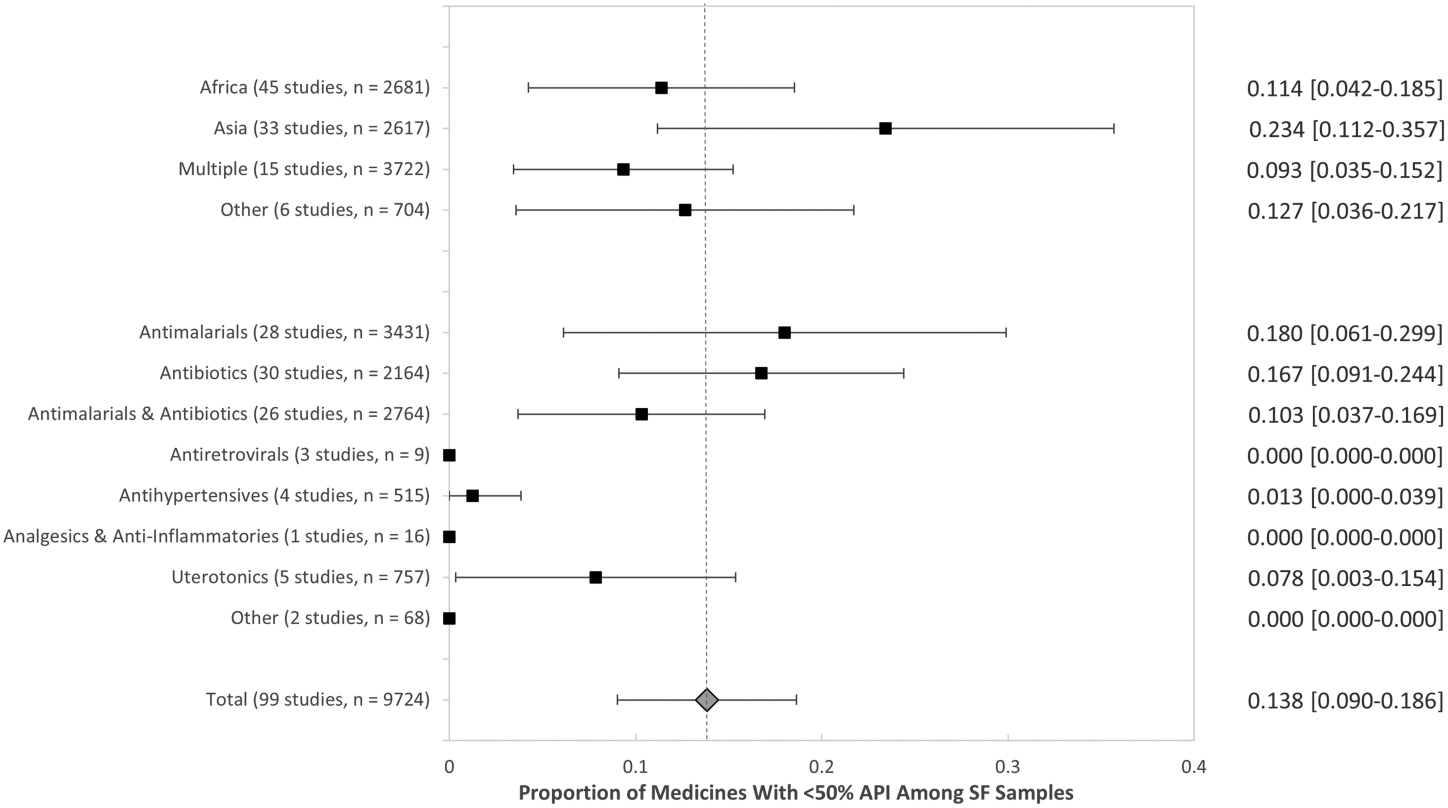
Medicines with < 50% active pharmaceutical ingredient (API) among samples that failed medicine quality tests. Sample size includes medicines found to be substandard or falsified across medicine quality studies. Classifications among therapeutic classes are the same as in Figure 2.

Figure 4 presents a subgroup analysis. The proportion of samples reported to contain < 50% API was highest in Asia at 23.4% (95% CI: 11.2–35.7%), compared with 12.7% (95% CI: 3.6–21.7%) for other single-region studies, 11.4% (95% CI: 4.2–18.5%) in Africa, and 9.3% (95% CI: 3.5–15.2%) in multiple-region studies. Across medicine samples reported to be substandard or falsified, antimalarials and antibiotics were most likely to be reported to contain < 50% API at 18.0% (95% CI: 6.1–29.9%) and 16.7% (95% CI: 9.1–24.4%), respectively. Studies that combined the results of antibiotics and antimalarials found 10.3% (95% CI: 3.7–16.9%) of samples contained < 50% API. Among poor-quality uterotonics, 7.8% (95% CI: 0.3–15.4%) were reported to contain < 50% API.

## DISCUSSION

Our results demonstrate that a quarter of the medicines that failed API quality tests in LMICs were reported to contain reduced API of < 80% of the stated amount. Only 12.5% of failed samples were found to have no API at all, an incorrect API, or both. This is an important finding because falsified medicines dealing with criminal activity tend to attract more attention than substandard medicines. Yet our results demonstrate that medicines with reduced API are also a pervasive problem, one that governments and policy makers need to allocate more resources toward combatting. Because both substandard and falsified medicines pose a threat to public health, it is critical to direct resources at them differently.

Our results provide some insight into where NMRAs should focus their attention. Medicines deviating from specifications with API < 80% were most commonly reported in Africa, followed by Asia. The prevalence of poor-quality analgesics and anti-inflammatories (46.8%) as well as uterotonics (46.6%), which are likely substandard, is alarming. Substandard medicines slightly deviating from standards can indicate, or are likely to arise from limited technical capacity, and deficient storage conditions at dispensing sites, where interventions should aim at ensuring sound practices.^3^^,^^121^ Substandard medicines can be reduced by strengthening Good Manufacturing Practices (GMP), Good Distribution Practices (GDP), Good Storage Practices (GSP), alongside medicine registration, prequalification of suppliers, and recalls.^122^^,^^123^

On the other hand, medicines reporting to contain < 50% API comprised a larger portion of poor-quality medicines in Asia (23.4%) compared with Africa (11.4%). Moreover, antimalarials and antibiotics were the therapeutic classes most likely reported to contain low API of < 50% API (18.0% and 16.7% of all poor-quality samples, respectively). This could be a sign of more falsification of these medicines, something that would require further testing and confirmation by NMRAs. Tackling falsified medicines requires coordination with law enforcement or customs authorities and may involve increased regulatory oversight, legal framework for prosecution, customs screening, post-market surveillance, and medication safety alerts. Falsification tends to flourish under high demand for medicines and poor governance, where criminals intend to make a profit.^24^^,^^124^ Therefore, preventing shortages or stock-outs and ensuring medication access are important parts of the solution.^3^

According to the WHO definition, a falsified medical product is one that intends to deceive.^6^ However, intention is difficult to assess. We found that most medicine quality studies do not report whether the product authenticity was confirmed by the manufacturer. Therefore, we used API amounts as a proxy to assess whether medicines are likely substandard or falsified. Although it is generally agreed upon that medicines with no or incorrect API are falsified,^11^ this cutoff would miss other falsified medicines intentionally manufactured with reduced amounts of API. We reasoned that medicines with < 50% API are likely to be falsified given that there was likely to be deliberate intent to make such medicines where no confirmation of intent was provided. In the absence of ability to confirm the intent to deceive, we consider that medicines containing < 50% API without evidence of decomposition is reasonable to denote likely falsification.^11^

Furthermore, we endorse the earlier call^11^ to improve reporting guidelines for medicine quality studies to distinguish substandard from falsified medicines (Table 2). Most medicines reported to be of poor-quality in LMICs did not specifically report the API amount of each sample. This makes our meta-analysis among samples reporting API levels conservative, because data were not available to classify every tested sample clearly and definitively. Currently, inconsistencies in reporting and combined results across countries, medicines, and sampled locations make it difficult to adequately assess risks and devise targeted interventions. We suggest that authors include exact API amounts rather than reporting only the number of samples that failed testing or API ranges, with further information on how and where those samples were obtained. We recommend that visual inspection, which can signal potential falsification,^125^ be accompanied by chemical testing to assess API amounts, along with an attempt to communicate with the manufacturer to confirm the original source. For medications with < 80% API, we suggest that studies report whether evidence of degradation exists to differentiate between samples that had degraded after manufacture and samples that were produced with insufficient API amounts. We also suggest that results of dissolution or disintegration tests be reported alongside API results when assessing the quality of tablets. Consistent and accurate reporting of medicine quality would not only aid in comparability of results but also inform countermeasures.

**Table 2:** Suggested guidelines for reporting poor-quality medicines as substandard or falsified medicines

Medicine quality	WHO definition	Operational characterizations of medicine quality	Suggested guidelines for medicine quality reporting
Falsified	Medical products that deliberately/fraudulently misrepresent their identity, composition or source	If at least one of the following is true: - Contains 0% - Contains an incorrect API - Manufacturer credibly confirms the packaging misrepresents the identity of the medicine - Analysis of the packaging gives conclusive evidence for falsification (e.g., the stated manufacturer does not exist)	Report numerical values of % API for every medicine tested, denoting the medicine, country, and region it was obtained from, sampled location (e.g., entry ports, warehouses, district hospitals, health centers, pharmacies, informal outlets), and method obtained (e.g., overt, mystery client). Visual inspection of packaging should be accompanied by findings from chemical testing to assess % API and results of communication with the manufacturer to confirm the source. Report if evidence for degradation exists (e.g., exhibiting multiple peaks in HPLC chromatogram) for samples containing < 80% API. Performance tests such as dissolution or disintegration test results should be reported for tablets alongside information on % API (e.g., results of Minilab tablet disintegration procedure).
Likely Falsified		Contains < 50% API and there is no evidence of decomposition	
Likely Substandard	Authorized medical products that fail to meet either their quality standards, specifications, or both	Extreme deviation^13^ - The content of API deviates by more than 20% from the declared content and/or - For tablets, an average dissolution value of tested units below pharmacopoeial Q value minus 25% Other deviations	

API = active pharmaceutical ingredient; HPLC = high-performance liquid chromatography.

There are several limitations to our analysis. First, systematic reviews are inherently limited by the search strategies used, databases searched, and inclusion and exclusion criteria applied. Our review update focused on PubMed as previous findings showed that few unique articles were identified from other databases.^9^ By cross-referencing with the Medicine Quality Scientific Literature Surveyor database, we believe we have captured the most pertinent literature. Second, meta-analyses are limited by the quality of included studies and the biases they contain.^126^ To minimize the impact of poor-quality studies in our analysis, we selected studies that tested 50 or more samples and weighted our meta-analyses by study sample size and MEDQUARG scores. Third, we observed considerable heterogeneity across medicine quality studies in reporting. For example, a considerable number of publications only reported API amounts below a cutoff rather than presenting a breakdown of actual API amounts of each sample. This prevented us from being able to develop mutually exclusive categories in our analysis. Our results for samples with 0% or < 50% API categories may be conservative because we were not able to assess the actual API amounts in some publications. Many publications were missing information on the criteria used to determine that a sample had failed. We suggest guidelines for reporting medicine quality studies to reduce reporting inconsistencies in the future. Lastly, our meta-analysis is likely influenced by publication bias where many studies are conducted in Africa and Asia testing antimalarials and antibiotics. Testing and reporting the quality of a wider range of medical products around the world will lend to a more comprehensive picture of the risks posed by substandard and falsified medicines. Despite these limitations, this meta-analysis offers a comprehensive and scientifically grounded method for differentiating poor-quality medicines across LMICs by reported API levels.

## CONCLUSION

This study contributes to the existing literature by providing an estimate of the magnitude of the problem of substandard and falsified medicines and examining the amounts of API in medicine samples that fail quality testing. Our findings of 12.4% overall prevalence of substandard and falsified medicines are consistent with previous analyses and WHO reports.^1^^,^^9^^,^^17^^,^^127^^–^^129^ Our analysis goes further by finding that nearly one in seven poor-quality medicine samples were likely to be falsified based on reported API amounts of < 50%, whereas the remaining six in seven samples were likely to be substandard. Separating out substandard from falsified medicines is essential to better inform tailored interventions to ensure medicine quality throughout the supply chain. Furthermore, we propose improved guidelines for reporting medicine quality in publications to better differentiate among poor-quality medicines. Governments and policy makers should use these results to target interventions to mitigate the threats of substandard and falsified medicines.

## Supplemental Material


Supplemental materials
