# Supplementary material for: Characterizing Medicine Quality by Active Pharmaceutical Ingredient Levels: A Systematic Review and Meta-Analysis across Low- and Middle-Income Countries
Source: Am J Trop Med Hyg. 2022 Jun 15;106(6):1778–90. doi: 10.4269/ajtmh.21-1123 (PMC9209904; doi:10.4269/ajtmh.21-1123)
Supplement: Supplementary file 1 [file tpmd211123.SD1.pdf]

Supplemental Appendix

Characterizing Medicine Quality By Active Pharmaceutical Ingredient Levels: A Systematic Review and Meta-Analysis Across Low- and Middle-Income Countries

Sachiko Ozawa[1,2*], Hui-Han Chen[1], Yi-Fang (Ashley) Lee[1], Colleen R. Higgins[1], Tatenda T. Yemeke[1]

[1]Division of Practice Advancement and Clinical Education, UNC Eshelman School of Pharmacy, University of North Carolina, Chapel Hill, NC, USA; [2]Department of Maternal and Child Health, UNC Gillings School of Global Public Health, University of North Carolina, Chapel Hill, NC, USA.

*Corresponding Author
Sachiko Ozawa
Associate Professor
Practice Advancement and Clinical Education
UNC Eshelman School of Pharmacy
University of North Carolina at Chapel Hill
Email: ozawa@unc.edu

Table of Contents

eAppendix 1: Comprehensive Search Strategy 2
eAppendix 2: Comprehensive List of 130 Studies Included in the Meta-analysis 4
Across Substandard and Falsified Samples 4
eAppendix 3: Forest Plot of Medicines with <50% API 12
eAppendix 4: MEDQUARG Scores and Interrater Reliability 13
eTable 1: MEDQUARG Scores of Studies of Second Search Included in the Meta-analysis 14
eAppendix 4.1 Interrater Reliability 15
eAppendix 5: Meta-analysis Bias and Quality Analysis 16
eAppendix 5.1 Heterogeneity: 16
eAppendix 5.2 Publication Bias 17
eFigure 1: Funnel Plot 17
eAppendix 5.3 Individual Study Influence on Heterogeneity 18
eFigure 2: Baujat Plot 18
eAppendix 5.4 Influence Plot and Output 19
eFigure 3: Influence Plot and Output 19
eAppendix 5.5 Moderating Effect 22

eAppendix 1: Comprehensive Search Strategy

Search for studies examining the prevalence of substandard and falsified medicines in LMICs

Conducted: February 4th, 2020

PubMed

Search	Query	Items Found
#1	("Counterfeit Drugs"[Mesh] OR ((counterfeit[tw] OR counterfeited[tw] OR counterfeits[tw] OR fake[tw] OR fakes[tw] OR faked[tw] OR falsified[tw] OR false[tw] OR counterfeiting[tw] OR substandard[tw] OR spurious[tw]) AND (medicine[tw] OR medicines[tw] OR medication[tw] OR medications[tw] OR drug[tw] OR drugs[tw] OR pharmaceutical[tw] OR pharmaceuticals[tw] OR "Pharmaceutical Preparations"[Mesh]))) NOT ("false positive"[tw] OR "false negative"[tw] OR "false transmitter"[tw] OR "false transmitters"[tw] OR "false result"[tw] OR "false results"[tw])	N/A
#2	((Deprived Countries[tw] OR Deprived Population[tw] OR Deprived Populations[tw] OR Developing Countries[tw] OR Developing Country[tw] OR Developing Economies[tw] OR Developing Economy[tw] OR Developing Nation[tw] OR Developing Nations[tw] OR Developing Population[tw] OR Developing Populations[tw] OR Developing World[tw] OR LAMI Countries[tw] OR LAMI Country[tw] OR Less Developed Countries[tw] OR Less Developed Country[tw] OR Less Developed Economies [tw] OR Less Developed Nation[tw] OR Less Developed Nations[tw] OR Less Developed World[tw] OR Lesser Developed Countries[tw] OR Lesser Developed Nations[tw] OR LMIC[tw] OR LMICS[tw] OR Low GDP[tw] OR Low GNP[tw] OR Low Gross Domestic[tw] OR Low Gross National[tw] OR Low Income Countries[tw] OR Low Income Country[tw] OR Low Income Economies [tw] OR Low Income Economy[tw] OR Low Income Nations[tw] OR Low Income Population[tw] OR Low Income Populations[tw] OR Lower GDP[tw] OR lower gross domestic[tw] OR Lower Income Countries[tw] OR Lower Income Country[tw] OR Lower Income Nations[tw] OR Lower Income Population[tw] OR Lower Income Populations[tw] OR Middle Income Countries[tw] OR Middle Income Country[tw] OR Middle Income Economies [tw] OR Middle Income Nation[tw] OR Middle Income Nations[tw] OR Middle Income Population[tw] OR Middle Income Populations[tw] OR Poor Countries[tw] OR Poor Country[tw] OR Poor Economies [tw] OR Poor Economy[tw] OR Poor Nation[tw] OR Poor Nations[tw] OR Poor Population[tw] OR Poor Populations[tw] OR poor world[tw] OR Poorer Countries[tw] OR Poorer Economies [tw] OR Poorer Economy[tw] OR Poorer Nations[tw] OR Poorer Population[tw] OR Poorer Populations[tw] OR Third World[tw] OR Transitional Countries[tw] OR Transitional Country[tw] OR Transitional Economies[tw] OR Transitional Economy[tw] OR Under Developed Countries[tw] OR Under Developed Country[tw] OR under developed nations[tw] OR Under Developed World[tw] OR Under Served Population[tw] OR Under Served Populations[tw] OR Underdeveloped Countries[tw] OR Underdeveloped Country[tw] OR underdeveloped economies[tw] OR underdeveloped nations[tw] OR underdeveloped population[tw] OR Underdeveloped World[tw] OR Underserved Countries[tw] OR Underserved Nations[tw] OR Underserved Population[tw] OR Underserved Populations[tw]))	N/A
#3	(Afghanistan[tw] OR Albania[tw] OR Algeria[tw] OR “American Samoa”[tw] OR Angola[tw]	N/A

	OR Armenia[tw] OR Azerbaijan[tw] OR Bangladesh[tw] OR Belarus[tw] OR Byelarus[tw] OR Belorussia[tw] OR Belize[tw] OR Benin[tw] OR Bhutan[tw] OR Bolivia[tw] OR Bosnia[tw] OR Botswana[tw] OR Brazil[tw] OR Bulgaria[tw] OR Burma[tw] OR "Burkina Faso"[tw] OR Burundi[tw] OR "Cabo Verde"[tw] OR "Cape verde"[tw] OR Cambodia[tw] OR Cameroon[tw] OR "Central African Republic"[tw] OR Chad[tw] OR China[tw] OR Colombia[tw] OR Comoros[tw] OR Comores[tw] OR Comoro[tw] OR Congo[tw] OR "Costa Rica"[tw] OR "Côte d'Ivoire"[tw] OR Cuba[tw] OR Djibouti[tw] OR Dominica[tw] OR "Dominican Republic"[tw] OR Ecuador[tw] OR Egypt[tw] OR "El Salvador"[tw] OR Eritrea[tw] OR Ethiopia[tw] OR Fiji[tw] OR Gabon[tw] OR Gambia[tw] OR Gaza[tw] OR "Georgia Republic"[tw] OR Georgian[tw] OR Ghana[tw] OR Grenada[tw] OR Grenadines[tw] OR Guatemala[tw] OR Guinea[tw] OR "Guinea Bisau"[tw] OR Guyana[tw] OR Haiti[tw] OR Herzegovina[tw] OR Hercegovina[tw] OR Honduras[tw] OR India[tw] OR Indonesia[tw] OR Iran[tw] OR Iraq[tw] OR Jamaica[tw] OR Jordan[tw] OR Kazakhstan[tw] OR Kenya[tw] OR Kiribati[tw] OR Korea[tw] OR Kosovo[tw] OR Kyrgyz[tw] OR OR Kirghizia[tw] OR Kirghiz[tw] OR Kirgizstan[tw] OR Kyrgyzstan[tw] OR "Lao PDR"[tw] OR Laos[tw] OR Lebanon[tw] OR Lesotho[tw] OR Liberia[tw] OR Libya[tw] OR Macedonia[tw] OR Madagascar[tw] OR Malawi[tw] OR Malay[tw] OR Malaya[tw] OR Malaysia[tw] OR Maldives[tw] OR Mali[tw] OR "Marshall Islands"[tw] OR Mauritania[tw] OR Mauritius[tw] OR Mexico[tw] OR Micronesia[tw] OR Moldova[tw] OR Mongolia[tw] OR Montenegro[tw] OR Morocco[tw] OR Mozambique[tw] OR Myanmar[tw] OR Namibia[tw] OR Nepal[tw] OR Nicaragua[tw] OR Niger[tw] OR Nigeria [tw] OR Pakistan [tw] OR Palau[tw] OR Panama[tw] OR "Papua New Guinea"[tw] OR Paraguay[tw] OR Peru [tw] OR Philippines[tw] OR Phillippines[tw] OR Philipines[tw] OR Phillipines[tw] OR Principe[tw] OR Romania[tw] OR Rwanda[tw] OR Ruanda[tw] OR Samoa[tw] OR "Sao Tome"[tw] OR Senegal[tw] OR Serbia[tw] OR "Sierra Leone"[tw] OR "Solomon Islands"[tw] OR Somalia[tw] OR "South Africa"[tw] OR "South Sudan"[tw] OR "Sri Lanka"[tw] OR "St Lucia"[tw] OR "St Vincent"[tw] OR Sudan[tw] OR Suriham[tw] OR Suriname[tw] OR Swaziland[tw] OR Syria[tw] OR "Syrian Arab Republic"[tw] OR Tajikistan[tw] OR Tadzhikistan[tw] OR Tadjikistan[tw] OR Tadzhik[tw] OR Tanzania[tw] OR Thailand[tw] OR Timor[tw] OR Togo[tw] OR Tonga[tw] OR Tunisia[tw] OR Turkey[tw] OR Turkmen[tw] OR Turkmenistan[tw] OR Tuvalu[tw] OR Uganda[tw] OR Ukraine[tw] OR Uzbek[tw] OR Uzbekistan[tw] OR Vanuatu[tw] OR Vietnam[tw] OR "West Bank"[tw] OR Yemen[tw] OR Zambia[tw] OR Zimbabwe[tw]))	
#4	#1 AND (#2 OR #3)	161

Total before duplicates removed: 161
Total after duplicates removed: 161

eAppendix 2: Comprehensive List of 130 Studies Included in the Meta-analysis Across Substandard and Falsified Samples

The 130 studies included in the meta-analysis across substandard and falsified samples are cited here (see **eReference** below), including 99 studies that were further included in the meta-analysis among samples tested for falsification,[1-99] and 31 studies that were not included in the meta-analysis among samples tested for falsification.[100-130]

eReference

1. Roy J, Saha P, Rahman A, Zakaria M. Quality of marketed paracetamol tablets in Bangladesh - An analytical overview. *Journal of Institute of Postgraduate Medicine and Research* 1993; **8**(2): 49-53.
2. Alotaibi N, Overton S, Curtis S, et al. Toward Point-of-Care Drug Quality Assurance in Developing Countries: Comparison of Liquid Chromatography and Infrared Spectroscopy Quantitation of a Small-Scale Random Sample of Amoxicillin. *The American journal of tropical medicine and hygiene* 2018; **99**(2): 477-81.
3. Bate R, Jin GZ, Mathur A. Counterfeit or Substandard? The Role of Regulation and Distribution Channel in Drug Safety. National Bureau of Economic Research, Inc, NBER Working Papers: 18073; 2012.
4. Bate R, Jensen P, Hess K, Mooney L, Milligan J. Substandard and falsified anti-tuberculosis drugs: a preliminary field analysis. *The international journal of tuberculosis and lung disease : the official journal of the International Union against Tuberculosis and Lung Disease* 2013; **17**(3): 308-11.
5. Bate R, Jin GZ, Mathur A, Attaran A. Poor Quality Drugs and Global Trade: A Pilot Study. National Bureau of Economic Research, Inc, NBER Working Papers: 20469; 2014.
6. Bate R, Mathur A. Corruption and medicine quality in Latin America: a pilot study. *The BE Journal of Economic Analysis & Policy* 2018; **18**(2).
7. Boadu RF, Agyare C, Adarkwa-Yiadom M, Adu F, Boamah VE, Boakye YD. In vitro Activity and Evaluation of Quality of Some Selected Penicillins on the Ghanaian Market using Developed HPLC Methods. 2015.
8. Exebio LEM, Rodríguez J, Sayritupac F. Los medicamentos falsificados en Perú. *Revista Panamericana de Salud Pública* 2010; **27**: 138-43.
9. Islam MR, Yoshida N, Kimura K, et al. An Investigation into the Quality of Medicines in Yangon, Myanmar. *Pharmacy (Basel, Switzerland)* 2018; **6**(3).
10. Kamau F, Thoithi G, Ngugi J, King'ondu O, Kibwage IO. Quality of amoxycillin preparations on the Kenyan market. *East and Central African Journal of Pharmaceutical Sciences* 2003; **6**(3): 57-60.
11. Khan MH, Hatanaka K, Sovannarith T, et al. Effects of packaging and storage conditions on the quality of amoxicillin-clavulanic acid - an analysis of Cambodian samples. *BMC pharmacology & toxicology* 2013; **14**: 33.
12. Khurelbat D, Dorj G, Bayarsaikhan E, et al. Prevalence estimates of substandard drugs in Mongolia using a random sample survey. *SpringerPlus* 2014; **3**: 709.
13. Khurelbat D, Dorj G, Sunderland B, et al. A cross-sectional analysis of falsified, counterfeit and substandard medicines in a low-middle income country. *BMC Public Health* 2020; **20**(1): 743.
14. Kumar P. A Study on Spurious and Not of Standard Quality Drugs in the State of Andhra Pradesh. *African Journal of Pharmacy and Pharmacology* 2018; **12**(10): 130-5.
15. Kitutu F. Screening drug quality project report. *Kampala: Uganda Medicines Transparency Alliance (MeTa)* 2015.

16. Laserson KF, Kenyon AS, Kenyon TA, Layloff T, Binkin NJ. Substandard tuberculosis drugs on the global market and their simple detection. *The international journal of tuberculosis and lung disease : the official journal of the International Union against Tuberculosis and Lung Disease* 2001; **5**(5): 448-54.
17. Lawal MG, Mukhtar MD, Magashi AM. Quality Assessment of Antibiotic Oral Drug Formulations Marketed In Katsina State, Nigeria. *Asian Journal of Pharmaceutical Research and Development* 2019; **7**(6): 6-10.
18. Myers NM, Maina MW, Were PM, et al. Lab on paper: assay of beta-lactam pharmaceuticals by redox titration. *Analytical Methods* 2019; **11**(37): 4741-50.
19. Nabirova D, Schmid G, Yusupova R, et al. Assessment of the quality of anti-tuberculosis medicines in Almaty, Kazakhstan, 2014. *The international journal of tuberculosis and lung disease : the official journal of the International Union against Tuberculosis and Lung Disease* 2017; **21**(10): 1161-8.
20. Nazerali H, Hogerzeil HV. The quality and stability of essential drugs in rural Zimbabwe: controlled longitudinal study. *BMJ (Clinical research ed)* 1998; **317**(7157): 512-3.
21. Obaid A. Quality of ceftriaxone in Pakistan: reality and resonance. *Pakistan journal of pharmaceutical sciences* 2009; **22**(2): 220-9.
22. Patel A, Gauld R, Norris P, Rades T. Quality of generic medicines in South Africa: Perceptions versus Reality–A qualitative study. *BMC health services research* 2012; **12**(1): 297.
23. Sabartova J, Nathanson E, Polishchuk O. Survey of the quality of anti-tuberculosis medicines circulating in selected newly independent states of the former Soviet Union. Geneva: World Health Organization; 2011.
24. Sakolchai S, Chaiyakum A, Wiyakrutta S, et al. A Survey on Qualities of Drugs Commercially Avilable in Thailand. *Srinagarind Medical Journal* 1991; **6**(3): 155-64.
25. Schäfermann S, Wemakor E, Hauk C, Heide L. Quality of medicines in southern Togo: Investigation of antibiotics and of medicines for non-communicable diseases from pharmacies and informal vendors. *PloS one* 2018; **13**(11): e0207911.
26. Tabernero P, Swamidoss I, Mayxay M, et al. A random survey of the prevalence of falsified and substandard antibiotics in the Lao PDR. *The Journal of antimicrobial chemotherapy* 2019; **74**(8): 2417-25.
27. Tshilumba PM, Amuri SB, Kaghowa ER, et al. Survey of some counterfeit anti-infective agents administered orally and marketed in the city of Lubumbashi. / Enquête sur la contrefaçon de quelques anti-infectieux administrés per os commercialisés dans la ville de Lubumbashi. *Pan African Medical Journal* 2015; **22**: 318-.
28. Wahidullah Karwar MZO, Zakeria Fatehzada, Aisha Noorzaee, Inua Yusuf, D. Lee, M. Morris, and T. Layloff. . Afghanistan Medicines Sampling and Testing – A Quantitative Survey. . *Submitted to the USAID by the Strengthening Pharmaceutical Systems (SPS) Program* 2011.
29. Wang T, Hoag SW, Eng ML, Polli J, Pandit NS. Quality of antiretroviral and opportunistic infection medications dispensed from developing countries and Internet pharmacies. *J Clin Pharm Ther* 2015; **40**(1): 68-75.
30. World Health Organization. Survey of the quality of medicines identified by the United Nations commission on life-saving commodities for women and children. 2016.
31. Yoshida N, Khan MH, Tabata H, et al. A cross-sectional investigation of the quality of selected medicines in Cambodia in 2010. *BMC pharmacology & toxicology* 2014; **15**: 13.
32. Antignac M, Diop BI, Macquart de Terline D, et al. Fighting fake medicines: First quality evaluation of cardiac drugs in Africa. *International journal of cardiology* 2017; **243**: 523-8.
33. Ndichu ET, Ohiri K, Sekoni O, Makinde O, Schulman K. Evaluating the quality of antihypertensive drugs in Lagos State, Nigeria. *PloS one* 2019; **14**(2): e0211567.
34. Rahman MS, Yoshida N, Tsuboi H, et al. A Cross-Sectional Investigation of the Quality of Selected Medicines for Noncommunicable Diseases in Private Community Drug Outlets in Cambodia during 2011-2013. *The American journal of tropical medicine and hygiene* 2019; **101**(5): 1018-26.

35. Redfern J, Kaur H, Adedoyin RA, et al. Equivalence in Active Pharmaceutical Ingredient of Generic Antihypertensive Medicines Available in Nigeria (EQUIMEDS): A Case for Further Surveillance. *Global heart* 2019; **14**(3): 327-33.
36. Amin AA, Snow RW, Kokwaro GO. The quality of sulphadoxine-pyrimethamine and amodiaquine products in the Kenyan retail sector. *J Clin Pharm Ther* 2005; **30**(6): 559-65.
37. Basco LK. Molecular epidemiology of malaria in Cameroon. XIX. Quality of antimalarial drugs used for self-medication. *The American journal of tropical medicine and hygiene* 2004; **70**(3): 245-50.
38. Belew S, Suleman S, Mohammed T, et al. Quality of fixed dose artemether/lumefantrine products in Jimma Zone, Ethiopia. *Malaria journal* 2019; **18**(1): 236.
39. Bjorkman Nyqvist M, Svensson J, Yanagizawa-Drott D. Can Good Products Drive Out Bad? Evidence from Local Markets for (Fake?) Antimalarial Medicine in Uganda. C.E.P.R. Discussion Papers, CEPR Discussion Papers: 9114; 2012.
40. Dondorp AM, Newton PN, Mayxay M, et al. Fake antimalarials in Southeast Asia are a major impediment to malaria control: multinational cross-sectional survey on the prevalence of fake antimalarials. *Tropical medicine & international health : TM & IH* 2004; **9**(12): 1241-6.
41. Evans L, 3rd, Coignez V, Barojas A, et al. Quality of anti-malarials collected in the private and informal sectors in Guyana and Suriname. *Malaria journal* 2012; **11**: 203.
42. Guo S, Kyaw MP, He L, et al. Quality Testing of Artemisinin-Based Antimalarial Drugs in Myanmar. *The American journal of tropical medicine and hygiene* 2017; **97**(4): 1198-203.
43. Idowu OA, Apalara SB, Lasisi AA. Assessment of quality of chloroquine tablets sold by drug vendors in Abeokuta, Nigeria. *Tanzania health research bulletin* 2006; **8**(1): 45-6.
44. Ioset JR, Kaur H. Simple field assays to check quality of current artemisinin-based antimalarial combination formulations. *PloS one* 2009; **4**(9): e7270.
45. Kaur H, Goodman C, Thompson E, et al. A nationwide survey of the quality of antimalarials in retail outlets in Tanzania. *PloS one* 2008; **3**(10): e3403.
46. Kaur H, Clarke S, Lalani M, et al. Fake anti-malarials: start with the facts. *Malaria journal* 2016; **15**: 86.
47. Khin C, Myint H, Thaung H, et al. Quality assessment of antimalarials in two border areas (Tamu and Muse). *Myanmar Health Sciences Research Journal* 2016; **28**(1): 48-52.
48. Lalani M, Kaur H, Mohammed N, et al. Substandard antimalarials available in Afghanistan: a case for assessing the quality of drugs in resource poor settings. *The American journal of tropical medicine and hygiene* 2015; **92**(6 Suppl): 51-8.
49. Maponga C, Ondari C, Organization WH. The quality of antimalarials: a study in selected African countries. 2003.
50. Mufusama JP, Ndjoko Ioset K, Feineis D, Hoellein L, Holzgrabe U, Bringmann G. Quality of the antimalarial medicine artemether - lumefantrine in 8 cities of the Democratic Republic of the Congo. *Drug testing and analysis* 2018; **10**(10): 1599-606.
51. Mziray S, Mwamwitwa K, Kisoma S, et al. Post marketing surveillance of anti-malarial medicines in Tanzania. *Pharm Regul Aff* 2017; **6**(1): 1-5.
52. Newton P, Proux S, Green M, et al. Fake artesunate in southeast Asia. *Lancet (London, England)* 2001; **357**(9272): 1948-50.
53. Newton PN, Fernandez FM, Plancon A, et al. A collaborative epidemiological investigation into the criminal fake artesunate trade in South East Asia. *PLoS medicine* 2008; **5**(2): e32.
54. Ochekpe NA, Agbowuro AA, Attah SE. Correlation of price and quality of medicines: Assessment of some artemisinin antimalarials in nigeria based on gphf minilab. *International Journal of Drug Development and Research* 2010; **2**(1): 211-8.
55. Ogwal-Okeng JW, Okello DO, Odyek O. Quality of oral and parenteral chloroquine in Kampala. *East African medical journal* 1998; **75**(12): 692-4.

56. Osei-Safo D, Agbonon A, Konadu DY, et al. Evaluation of the quality of artemisinin-based antimalarial medicines distributed in ghana and togo. *Malar Res Treat* 2014; **2014**: 806416.
57. Phanouvong S, Raymond C, Krech L, et al. The quality of antimalarial medicines in western Cambodia: a case study along the Thai-Cambodian border. *The Southeast Asian journal of tropical medicine and public health* 2013; **44**(3): 349-62.
58. Tabernero P, Mayxay M, Culzoni MJ, et al. A Repeat Random Survey of the Prevalence of Falsified and Substandard Antimalarials in the Lao PDR: A Change for the Better. *The American journal of tropical medicine and hygiene* 2015; **92**(6 Suppl): 95-104.
59. Tipke M, Diallo S, Coulibaly B, et al. Substandard anti-malarial drugs in Burkina Faso. *Malaria journal* 2008; **7**: 95.
60. Visser BJ, Meerveld-Gerrits J, Kroon D, et al. Assessing the quality of anti-malarial drugs from Gabonese pharmacies using the MiniLab(R): a field study. *Malaria journal* 2015; **14**: 273.
61. World Health Organization. Survey of the quality of selected antimalarial medicines circulating in Madagascar, Senegal, and Uganda. November; 2009.
62. World Health Organization. Survey of the quality of selected antimalarial medicines circulating in six countries of sub-Saharan Africa. Geneva, Switzerland: World Health Organization, 2011.
63. Yeung S, Lawford HLS, Tabernero P, et al. Quality of antimalarials at the epicenter of antimalarial drug resistance: results from an overt and mystery client survey in Cambodia. *The American journal of tropical medicine and hygiene* 2015; **92**(6 Suppl): 39-50.
64. Baratta F, Germano A, Brusa P. Diffusion of counterfeit drugs in developing countries and stability of galenics stored for months under different conditions of temperature and relative humidity. *Croatian medical journal* 2012; **53**(2): 173-84.
65. Bate R, Mooney L, Hess K. Medicine registration and medicine quality: a preliminary analysis of key cities in emerging markets. *Research and Reports in Tropical Medicine* 2010; **1**: 89-93.
66. CDSCO MoHaFW, Government of India. Report on countrywide survey for spurious drugs. 2009.
67. Food and Drug Department, Food and Drug Quality Control Center. Country Report on Medicines Quality Monitoring Program in Laos (2005-2009). 2010. http://www.fdd.gov.la/download/contents_documents/1407835610country%20report%20in%20MQM%20progam%202005-2009.pdf (accessed December 03 2020).
68. Food and Drug Department, Food and Drug Quality Control Center. Comparative Study of the Quality, Availability, and Source of Antimalarial Medicines in Cambodia, Laos, Thailand, and Vietnam in PQM-MQM Covered and Non-Covered Areas in Mekong Sub-Region. 2014. http://www.fdd.gov.la/download/contents_documents/1411697106Lao%20Comparative%20Study%20Final.pdf (accessed December 03 2020).
69. Frimpong G, Ofori-Kwakye K, Kuntworbe N, et al. Quality Assessment of Some Essential Children's Medicines Sold in Licensed Outlets in Ashanti Region, Ghana. *Journal of tropical medicine* 2018; **2018**: 1494957.
70. Hajjou M, Krech L, Lane-Barlow C, et al. Monitoring the quality of medicines: results from Africa, Asia, and South America. *The American journal of tropical medicine and hygiene* 2015; **92**(6 Suppl): 68-74.
71. Hetzel MW, Page-Sharp M, Bala N, et al. Quality of antimalarial drugs and antibiotics in Papua New Guinea: a survey of the health facility supply chain. *PloS one* 2014; **9**(5): e96810.
72. Kaale E, Manyanga V, Chambuso M, et al. The Quality of Selected Essential Medicines Sold in Accredited Drug Dispensing Outlets and Pharmacies in Tanzania. *PloS one* 2016; **11**(11): e0165785.
73. Khan MH, Okumura J, Sovannarith T, et al. Counterfeit medicines in Cambodia--possible causes. *Pharmaceutical research* 2011; **28**(3): 484-9.

74. Khuluza F, Kigera S, Heide L. Low Prevalence of Substandard and Falsified Antimalarial and Antibiotic Medicines in Public and Faith-Based Health Facilities of Southern Malawi. *The American journal of tropical medicine and hygiene* 2017; **96**(5): 1124-35.
75. Kibwage I, Thuranira J, Gathu L, et al. Drug Quality Control Work in Drug Analysis and Research Unit: Observation During 1991-1995. *The East and Central African Journal of Pharmaceutical Sciences* 1999; **2**(2): 32-6.
76. Lon CT, Tsuyuoka R, Phanouvong S, et al. Counterfeit and substandard antimalarial drugs in Cambodia. *Transactions of the Royal Society of Tropical Medicine and Hygiene* 2006; **100**(11): 1019-24.
77. Petersen A, Held N, Heide L, Difam EPNMSG. Surveillance for falsified and substandard medicines in Africa and Asia by local organizations using the low-cost GPHF Minilab. *PloS one* 2017; **12**(9): e0184165.
78. Phanouvong S, Dijiba Y, Vijaykadga S, et al. The quality of antimalarial medicines in eastern Thailand: a case study along the Thai-Cambodian border. *The Southeast Asian journal of tropical medicine and public health* 2013; **44**(3): 363-73.
79. Pribluda VS, Barojas A, Anez A, et al. Implementation of basic quality control tests for malaria medicines in Amazon Basin countries: results for the 2005-2010 period. *Malaria journal* 2012; **11**: 202.
80. Risha PG, Msuya Z, Clark M, Johnson K, Ndomondo-Sigonda M, Layloff T. The use of Minilabs to improve the testing capacity of regulatory authorities in resource limited settings: Tanzanian experience. *Health policy (Amsterdam, Netherlands)* 2008; **87**(2): 217-22.
81. Schiavetti B, Wynendaele E, De Spiegeleer B, et al. The quality of medicines used in children and supplied by private pharmaceutical wholesalers in Kinshasa, Democratic Republic of Congo: a prospective survey. *The American journal of tropical medicine and hygiene* 2018; **98**(3): 894-903.
82. Seear M, Gandhi D, Carr R, Dayal A, Raghavan D, Sharma N. The need for better data about counterfeit drugs in developing countries: a proposed standard research methodology tested in Chennai, India. *J Clin Pharm Ther* 2011; **36**(4): 488-95.
83. Shakoor O, Taylor RB, Behrens RH. Assessment of the incidence of substandard drugs in developing countries. *Tropical medicine & international health : TM & IH* 1997; **2**(9): 839-45.
84. Stenson B, Lindgren BH, Syhakhang L, Tomson G. The quality of drugs in private pharmacies in the Lao People's Democratic Republic. *International Journal of Risk and Safety in Medicine* 1998; **11**(4): 243-9.
85. Syhakhang L. The quality of private pharmacy services in a province of Lao PDR: perceptions, practices and regulatory enforcements. Stockholm; Sweden: Karolinksa Institutet, Division of International Health, Department of Public Health Sciences; 2002.
86. Taylor RB, Shakoor O, Behrens RH, et al. Pharmacopoeial quality of drugs supplied by Nigerian pharmacies. *Lancet (London, England)* 2001; **357**(9272): 1933-6.
87. Alliance. UMT. Screening Drug Quality Project Report. Kampala, Uganda: Uganda Medicines Transparency Alliance. 2014.
88. Wondemagegnehu E. Counterfeit and substandard drugs in Myanmar and Viet Nam. *WHO Report WHO/EDM/QSM* 1999; **99**.
89. WHO Action Programme on Essential Drugs. La Qualité des médicaments sur le marché pharmaceutique africain : étude analytique dans trois pays, Cameroun, Madagascar, Tchad. Geneve, Switzerland: World Health Organization, 1995.
90. Kuwana R, Sabartova J. Survey of the quality of selected antiretroviral medicines circulating in five African countries. *WHO Drug Information* 2017; **31**(2): 162.
91. Ministry of Medical Services, Ministry of Public Health and Sanitation. Post market survey of antiretroviral medicines in Kenya. 2012. https://pharmacyboardkenya.org/files/?file=ARV%20Report%20Final%202012.pdf (accessed December 03 2020).

92. World Health Organization. Survey of the Quality of Antiretroviral Medicines Circulating in Selected African Countries. 2007.
93. Anyakora C, Oni Y, Ezedinachi U, et al. Quality medicines in maternal health: results of oxytocin, misoprostol, magnesium sulfate and calcium gluconate quality audits. *BMC pregnancy and childbirth* 2018; **18**(1): 44.
94. Hall PE. Quality of medicines: Quality of misoprostol products. *WHO Drug Information* 2016; **30**(1): 35-9.
95. Karikari-Boateng E, Ghana F, BOATENG KP. Post-Market Quality Surveillance Project Maternal Health Care Products (Oxytocin and Ergometrine) on the Ghanaian Market. *Accra, Ghana: Ghana Food and Drugs Authority* 2013.
96. Stanton C, Koski A, Cofie P, Mirzabagi E, Grady BL, Brooke S. Uterotonic drug quality: an assessment of the potency of injectable uterotonic drugs purchased by simulated clients in three districts in Ghana. *BMJ open* 2012; **2**(3).
97. Stanton C, Nand DN, Koski A, et al. Accessibility and potency of uterotonic drugs purchased by simulated clients in four districts in India. *BMC pregnancy and childbirth* 2014; **14**(1): 386.
98. Laroche ML, Traore H, Merle L, Gaulier JM, Viana M, Preux PM. Quality of phenobarbital solid-dosage forms in the urban community of Nouakchott (Mauritania). *Epilepsia* 2005; **46**(8): 1293-6.
99. Suleman S, Zeleke G, Deti H, et al. Quality of medicines commonly used in the treatment of soil transmitted helminths and giardia in ethiopia: a nationwide survey. *PLoS neglected tropical diseases* 2014; **8**(12): e3345.
100. Abdo-Rabbo A, Bassili A, Atta H. The quality of antimalarials available in Yemen. *Malaria journal* 2005; **4**: 28.
101. Act Consortium Drug Quality Project T, The Impact Study T. Quality of Artemisinin-Containing Antimalarials in Tanzania's Private Sector--Results from a Nationally Representative Outlet Survey. *The American journal of tropical medicine and hygiene* 2015; **92**(6 Suppl): 75-86.
102. Chikowe I, Osei-Safo D, Harrison JJ, Konadu DY, Addae-Mensah I. Post-marketing surveillance of anti-malarial medicines used in Malawi. *Malaria journal* 2015; **14**: 127.
103. Fotiou F, Aravind S, Wang PP, Nerapusee O. Impact of illegal trade on the quality of epoetin alfa in Thailand. *Clinical therapeutics* 2009; **31**(2): 336-46.
104. Gimenez F, Bruneton C, Rith DJMEMI. Quality assessment of drugs sold and delivered in Cambodia. 1997; **27**: 541-4.
105. Hadi U, van den Broek P, Kolopaking EP, Zairina N, Gardjito W, Gyssens IC. Cross-sectional study of availability and pharmaceutical quality of antibiotics requested with or without prescription (Over The Counter) in Surabaya, Indonesia. *BMC infectious diseases* 2010; **10**: 203.
106. Kenyan Ministry of Public Health and Sanitation. Monitoring the Quality of Antimalarial Medicines Circulating in Kenya. Nairobi, Kenya: Ministry of Public Health and Sanitation, 2011.
107. Kenyan Ministry of Public Health and Sanitation. Monitoring the Quality of Antimalarial Medicines Circulating in Kenya. Nairobi, Kenya: Ministry of Public Health and Sanitation, 2012.
108. Kyriacos S, Mroueh M, Chahine RP, Khouzam O. Quality of amoxicillin formulations in some Arab countries. *J Clin Pharm Ther* 2008; **33**(4): 375-9.
109. Mbaziira N. Registration and Quality Assurance of ARVs & Other Essential Medicines in Namibia: October 2014-September 2015. Arlington, VA: Management Sciences for Health, 2015.
110. Ogwal-Okeng JW, Owino E, Obua C. Chloroquine in the Ugandan market fails quality test: a pharmacovigilance study. *African health sciences* 2003; **3**(1): 2-6.
111. Okumura J, Taga M, Tey S, Kataoka Y, Nam N, Kimura K. High failure rate of the dissolution tests for 500-mg amoxicillin capsules sold in Cambodia: is it because of the product or the test method? *Tropical medicine & international health : TM & IH* 2010; **15**(11): 1340-6.

112. Onwujekwe O, Kaur H, Dike N, et al. Quality of anti-malarial drugs provided by public and private healthcare providers in south-east Nigeria. *Malaria journal* 2009; **8**: 22.
113. Pouillot R, Bilong C, Boisier P, et al. [Illicit drug trade on the markets of Yaounde (Cameroon) and Niamey (Niger): characteristics of salesmen and quality of drugs]. *Bulletin de la Societe de pathologie exotique (1990)* 2008; **101**(2): 113-8.
114. Ramachandran G, Chandrasekaran V, Hemanth Kumar AK, Dewan P, Swaminathan S, Thomas A. Estimation of content of anti-TB drugs supplied at centres of the Revised National TB Control Programme in Tamil Nadu, India. *Tropical medicine & international health : TM & IH* 2013; **18**(9): 1141-4.
115. Rookkapan K, Chongsuvivatwong V, Kasiwong S, Pariyawatee S, Kasetcharoen Y, Pungrassami P. Deteriorated tuberculosis drugs and management system problems in lower southern Thailand. *The international journal of tuberculosis and lung disease : the official journal of the International Union against Tuberculosis and Lung Disease* 2005; **9**(6): 654-60.
116. Sheth PD, Reddy M, Regal B, Kaushal M, Sen K, Narayana D. Extent of spurious (counterfeit) medicines in India. New Delhi: SEARPharm Forum in collaboration with Delhi Pharmaceutical Trust and Apothecaries Foundation for WHO; 2007; 2007.
117. Syhakhang L, Lundborg CS, Lindgren B, Tomson G. The quality of drugs in private pharmacies in Lao PDR: a repeat study in 1997 and 1999. *Pharmacy world & science : PWS* 2004; **26**(6): 333-8.
118. Tivura M, Asante I, van Wyk A, et al. Quality of Artemisinin-based Combination Therapy for malaria found in Ghanaian markets and public health implications of their use. *BMC pharmacology & toxicology* 2016; **17**(1): 48.
119. Vijaykadga S, Cholpol S, Sitthimongkol S, et al. Strengthening of national capacity in implementation of antimalarial drug quality assurance in Thailand. *The Southeast Asian journal of tropical medicine and public health* 2006; **37 Suppl 3**: 5-10.
120. Wafula F, Dolinger A, Daniels B, et al. Examining the Quality of Medicines at Kenyan Healthcare Facilities: A Validation of an Alternative Post-Market Surveillance Model That Uses Standardized Patients. *Drugs - real world outcomes* 2017; **4**(1): 53-63.
121. Yang D, Plianbangchang P, Visavarungroj N, Rujivipat S. Quality of pharmaceutical items available from drugstores in Phnom Penh, Cambodia. *The Southeast Asian journal of tropical medicine and public health* 2004; **35**(3): 741-7.
122. Chen H, Tan C, Lin Z. Express detection of expired drugs based on near-infrared spectroscopy and chemometrics: A feasibility study. *Spectrochimica acta Part A, Molecular and biomolecular spectroscopy* 2019; **220**: 117153.
123. Jost J, Ratsimbazafy V, Nguyen TT, et al. Quality of antiepileptic drugs in sub-Saharan Africa: A study in Gabon, Kenya, and Madagascar. *Epilepsia* 2018; **59**(7): 1351-61.
124. Kakio T, Nagase H, Takaoka T, et al. Survey to Identify Substandard and Falsified Tablets in Several Asian Countries with Pharmacopeial Quality Control Tests and Principal Component Analysis of Handheld Raman Spectroscopy. *The American journal of tropical medicine and hygiene* 2018; **98**(6): 1643-52.
125. Lawson G, Ogwu J, Tanna S. Quantitative screening of the pharmaceutical ingredient for the rapid identification of substandard and falsified medicines using reflectance infrared spectroscopy. *PloS one* 2018; **13**(8): e0202059.
126. Lehmann A, Katerere DR, Dressman J. Drug quality in South Africa: a field test. *Journal of pharmaceutical sciences* 2018; **107**(10): 2720-30.
127. Macquart de Terline D, Diop BI, Bernard M, et al. Substandard drugs among five common antihypertensive generic medications: an analysis from 10 African countries. *Journal of hypertension* 2018; **36**(2): 395-401.

128. Dirección General de Medicamentos Insumos y Drogas. Iniciativa de Enfermedades Infecciosas en América del Sur: Resumen del Segundo Estudio para Determinar la Calidad de Antimicrobianos y Antituberculosos Utilizados en la Red BEPECA de la Dirección de Salud Callao, 2009.
129. Krech LA, Lane-Barlow C, Lang S, et al. Cambodian ministry of health takes decisive actions in the fight against substandard and counterfeit medicines. *Tropical Medicine Surgery* 2014.
130. Ratanawijitrasin S, Phanouvong S. The state of medicine quality in the Mekong sub-region: Institut de recherche sur l'Asie du Sud-Est contemporaine; 2018.
131. Newton PN, Lee SJ, Goodman C, et al. Guidelines for field surveys of the quality of medicines: a proposal. *PLoS medicine* 2009; **6**(3): e52.
132. Almuzaini T, Choonara I, Sammons H. Substandard and counterfeit medicines: a systematic review of the literature. *BMJ open* 2013; **3**(8): e002923.
133. Ozawa S, Evans DR, Bessias S, et al. Prevalence and Estimated Economic Burden of Substandard and Falsified Medicines in Low- and Middle-Income Countries: A Systematic Review and Meta-analysis. *JAMA network open* 2018; **1**(4): e181662.

eAppendix 3: Forest Plot of Medicines with <50% API

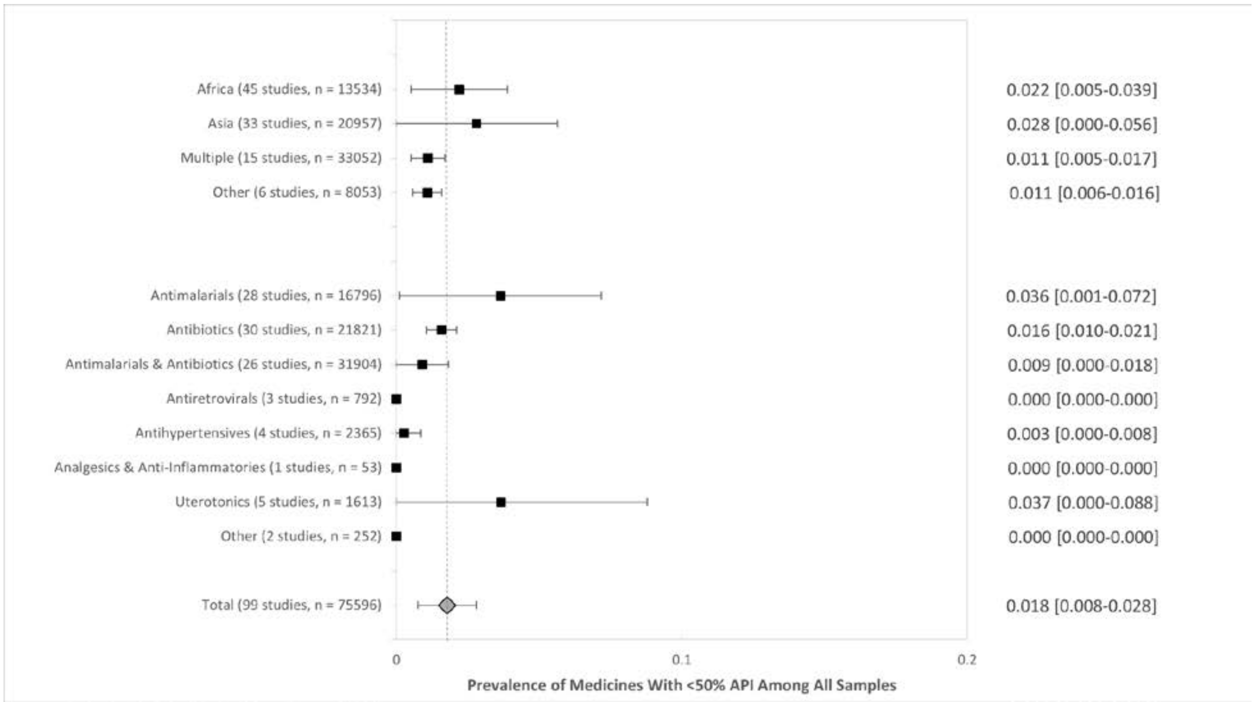

Note: Sample size includes studies with enough information to distinguish medicines containing <50% API. Antimalarials include studies that examined antimalarials but not antibiotics. Antibiotics include studies that examined antibiotics but not antimalarials. Antimalarials & antibiotics include studies that examined both antimalarials and antibiotics. Sample sizes of (1) antiretrovirals, (2) antihypertensives, (3) analgesics & anti-inflammatories, and (4) uterotonics includes studies that investigated the specific therapeutic category but not antibiotics or antimalarials, and may or may not include other therapeutic categories.

eAppendix 4: MEDQUARG Scores and Interrater Reliability

In recent years, the Medicine Quality Assessment Reporting Guidelines (MEDQUARG) has been used to ensure the quality of research assessing the quality of drugs.[131] A 12-item of checklist was proposed by Almuzaini et al to examine the quality of publication based on the MEDQUARG criteria.[132] Each publication is assigned a score based on how many items the study meets the 12-item checklist. The 12-item criteria are shown below:

1. Timing and location of study clearly stated.
2. Definition of counterfeit or substandard medicines used mentioned.
3. Type of outlets sampled.
4. Sampling design and sample size calculation described.
5. Type and number of dosage units purchased per outlet.
6. Random sampling used.
7. Information on who collected the samples (were mystery shoppers applied?)
8. Packaging assessment performed.
9. Statistical analysis described.
10. Chemical analysis clearly described.
11. Details on method validation.
12. Chemical analysis performed blinded to packaging.

We evaluated the quality of each publication and assigned a MEDQUARG score to the publication based on the 12-item checklist. Each study was examined by two reviewers independently. If any discrepancy was raised between the two reviewers, the reviewers met and discussed about situation and assigned a final score to the publication. To ensure the interrater reliability between the two reviewers and between each reviewer and the final score, we calculated the Spearman's correlation coefficient between the scores. The result demonstrated a significant interrater reliability between two reviewers.

eTable 1: MEDQUARG Scores of Studies of Second Search Included in the Meta-analysis

Author (Year)	MEDQUARG First rater	MEDQUARG Second rater	FINAL
Alotaibi et al.[2] (2018)	7	6	6
Anyakora et al.[93] (2018)	8	8	8
Bate et al.[6] (2018)	7	7	7
Belew et al.[38] (2019)	9	7	8
Boadu et al.[7] (2015)	8	8	8
Chen et al.[122] (2019)	5	4	4
DIGEMID[128] (2009)	4	5	4
Exebio et al.[8] (2010)	3	3	3
FDD[67] (2010)	6	4	5
FDD[68] (2014)	8	7	7
Frimpong et al.[69] (2018)	6	7	6
Islam et al.[9] (2018)	8	7	7
Jost et al.[123] (2018)	7	6	6
Kakio et al.[124] (2018)	6	7	6
Kamau et al.[10] (2003)	2	2	2
Khurelbat et al.[13] (2020)	9	10	9
Kibwage et al.[75] (1999)	3	3	3
Krech et al.[129] (2014)	6	5	5
Kumar et al.[14] (2018)	1	1	1
Lawal et al.[17] (2019)	3	4	3
Lawson et al.[125] (2018)	4	3	3
Lehmann et al.[126] (2018)	11	12	11
Mufusama et al.[50] (2018)	5	6	5
Myers et al.[18] (2019)	4	5	4
Mziray et al.[51] (2017)	8	5	6
MOMS[91] (2012)	7	4	5
Ndichu et al.[33] (2019)	7	8	7
Rahman et al.[34] (2019)	10	10	10
Ratanawijitrasin et al.[130] (2014)	6	6	6
Redfern et al.[35] (2019)	6	4	5
Sakolkhai et al.[24] (1991)	3	3	3
Schafermann et al.[25] (2018)	9	10	9
Schiavetti et al.[81] (2018)	10	10	10
Tabernero et al.[26] (2019)	8	9	8
Terline et al.[127] (2018)	7	9	8
WHO[89] (1995)	5	4	4

Footnote: this table listed the 36 new studies found in the second search. The MEDQUARG scores of the other 94 studies can be found in the Ozawa et al (2018).[133]

eAppendix 4.1 Interrater Reliability

1. Spearman correlation between the two reviewers

```
. spearman First Second

 Number of obs =      36
Spearman's rho =       0.8730

Test of Ho: First and Second are independent
    Prob > |t| =       0.0000
```

2. Spearman correlation between the first reviewer and the final score

```
. spearman First Final

 Number of obs =      36
Spearman's rho =       0.9582

Test of Ho: First and Final are independent
    Prob > |t| =       0.0000
```

3. Spearman correlation between the second reviewer and the final score

```
. spearman Second Final

 Number of obs =      36
Spearman's rho =       0.9649

Test of Ho: Second and Final are independent
    Prob > |t| =       0.0000
```

eAppendix 5: Meta-analysis Bias and Quality Analysis

To access the heterogeneity of studies included in the meta-analysis, we conducted a random effects model for weighted point estimates. The heterogeneity of the analysis was performed using Cochran's Q and I^2. We examined the publication bias via a funnel plot analysis. Influence of individual studies was assessed using both Baujat plot and influence plot. Five potential moderators were tested to determine the potential sources of heterogeneity. The moderators included MEDGUARG score, publication year, number of samples tested, medicine category, and region.

Heterogeneity: The random effects model showed a significant heterogeneity of the studies included in the meta-analysis. The Q value was significant and the I^2 (99.92%) indicated a significant amount of heterogeneity across the studies.

Influence: Baujat and influence plots showed that study 15 (Chikowe et al., 2015)[102], 73 (Stanton et al., 2012)[96], and 94 (Yang et al., 2004)[121] had large amount of influence on the meta-analysis. These three studies reported that more than 88% of the samples tested were identified poor-quality.

Publication Bias: The funnel plot showed asymmetry distribution of the studies. Studies with lower standard errors reported smaller proportion of the samples tested. The mixed-effects meta-regression result showed a significant publication bias (P < 0.0001).

Moderators: Number of samples tested and region were found as significant moderator to the heterogeneity.

eAppendix 5.1 Heterogeneity:

```
Random-Effects Model (k = 130; tau^2 estimator: REML)

tau^2 (estimated amount of total heterogeneity): 0.0422 (SE = 0.0054)
tau (square root of estimated tau^2 value):      0.2053
I^2 (total heterogeneity / total variability):   99.92%
H^2 (total variability / sampling variability):  1325.60

Test for Heterogeneity:
Q(df = 129) = 17099.7522, p-val < .0001

Model Results:

estimate      se     zval    pval   ci.lb   ci.ub
  0.2388  0.0182  13.1114  <.0001  0.2031  0.2745  ***

---
Signif. codes:  0 ‘***’ 0.001 ‘**’ 0.01 ‘*’ 0.05 ‘.’ 0.1 ‘ ’ 1

        estimate     ci.lb     ci.ub
tau^2     0.0422    0.0333    0.0550
tau       0.2053    0.1825    0.2345
I^2(%)   99.9246   99.9046   99.9422
```

eAppendix 5.2 Publication Bias:

eFigure 1: Funnel Plot

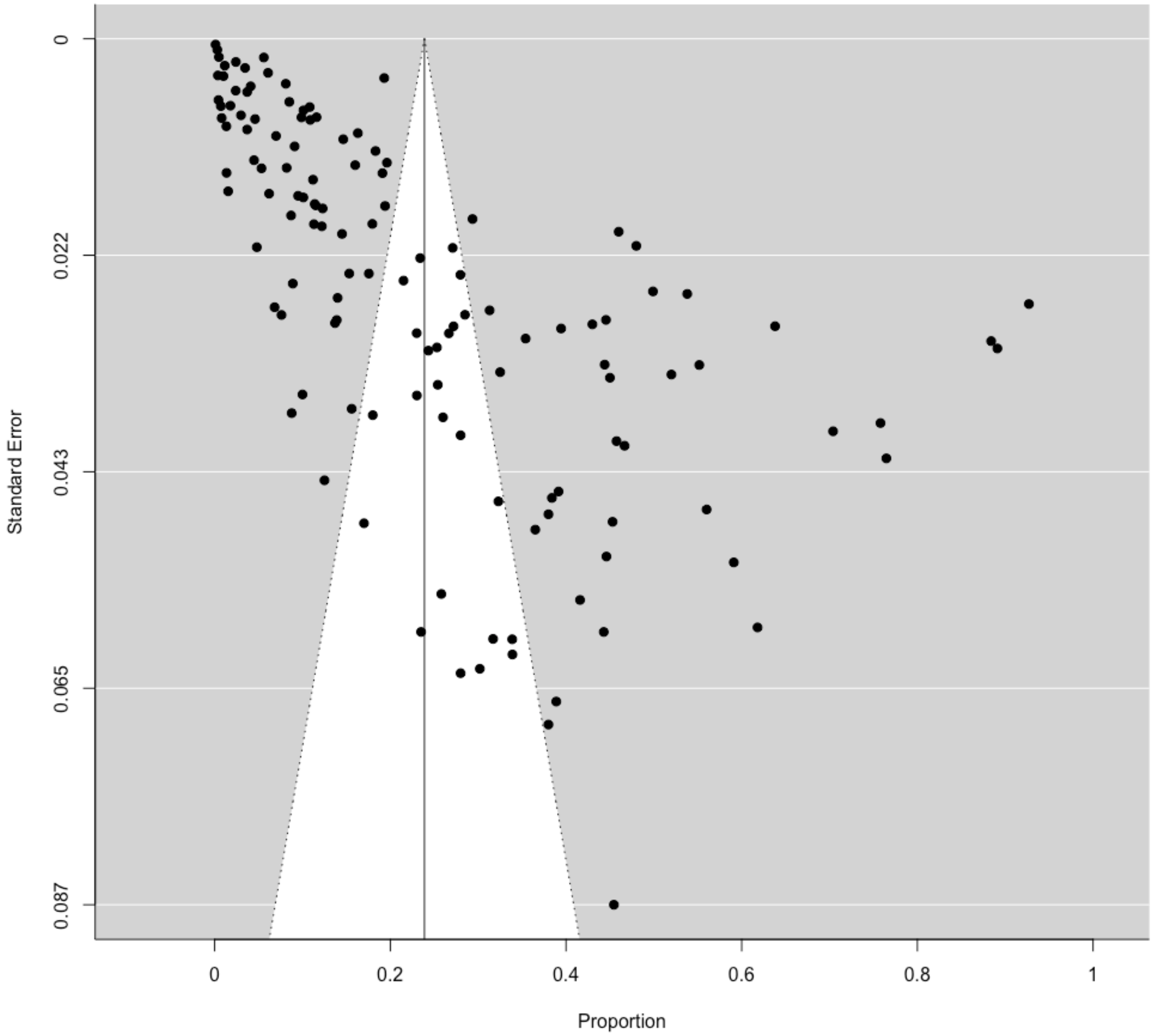


```
Regression Test for Funnel Plot Asymmetry

model:     mixed-effects meta-regression model
predictor: standard error

test for funnel plot asymmetry: z = 8.5508, p < .0001
```

eAppendix 5.3 Individual Study Influence on Heterogeneity:

eFigure 2: Baujat Plot

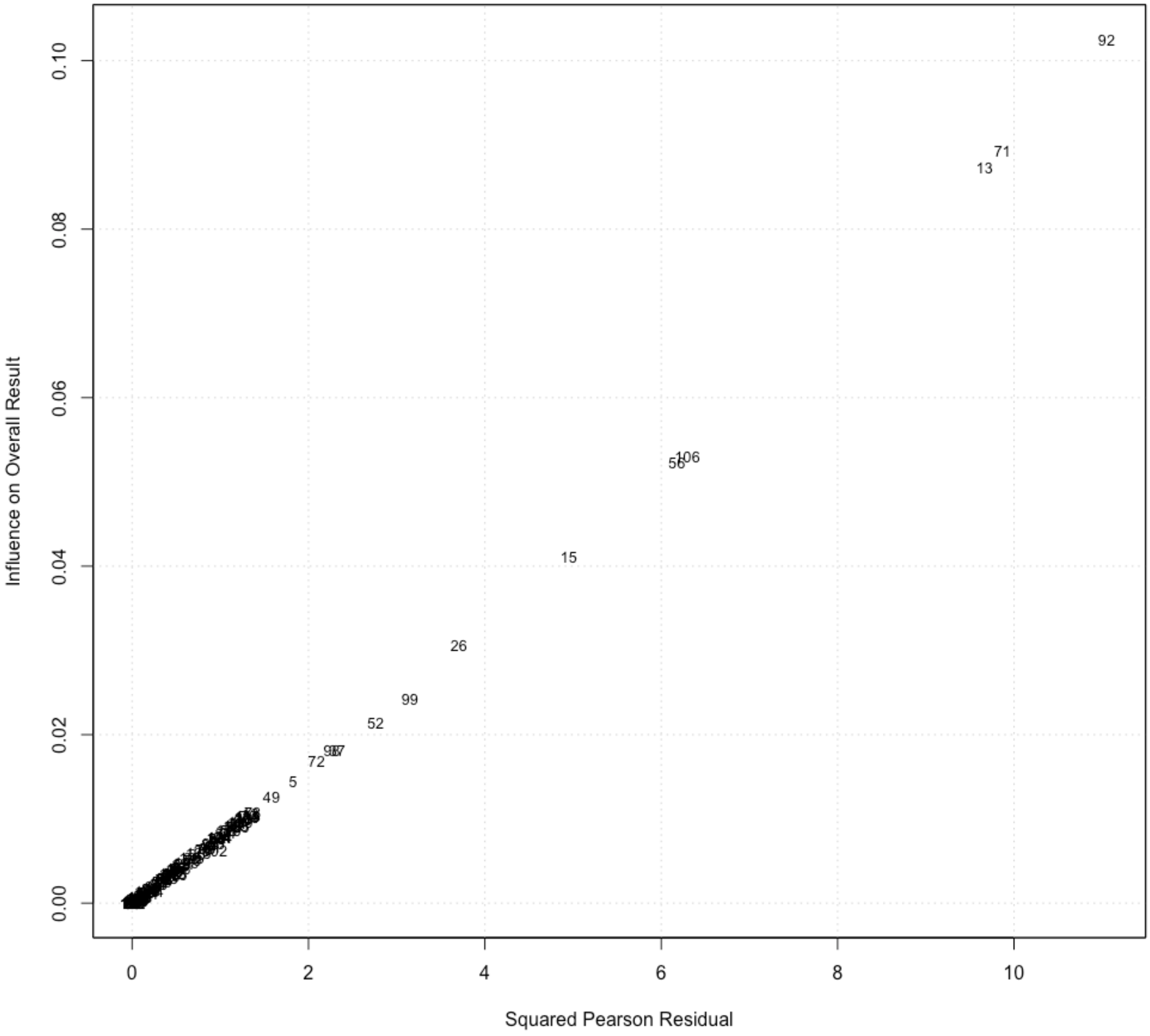

*Footnote: study 15 (Chikowe et al., 2015)[102], 73 (Stanton et al., 2012)[96], and 94 (Yang et al., 2004)[121] had large amount of influence on the meta-analysis.

eAppendix 5.4 Influence Plot and Output:

eFigure 3: Influence Plot and Output

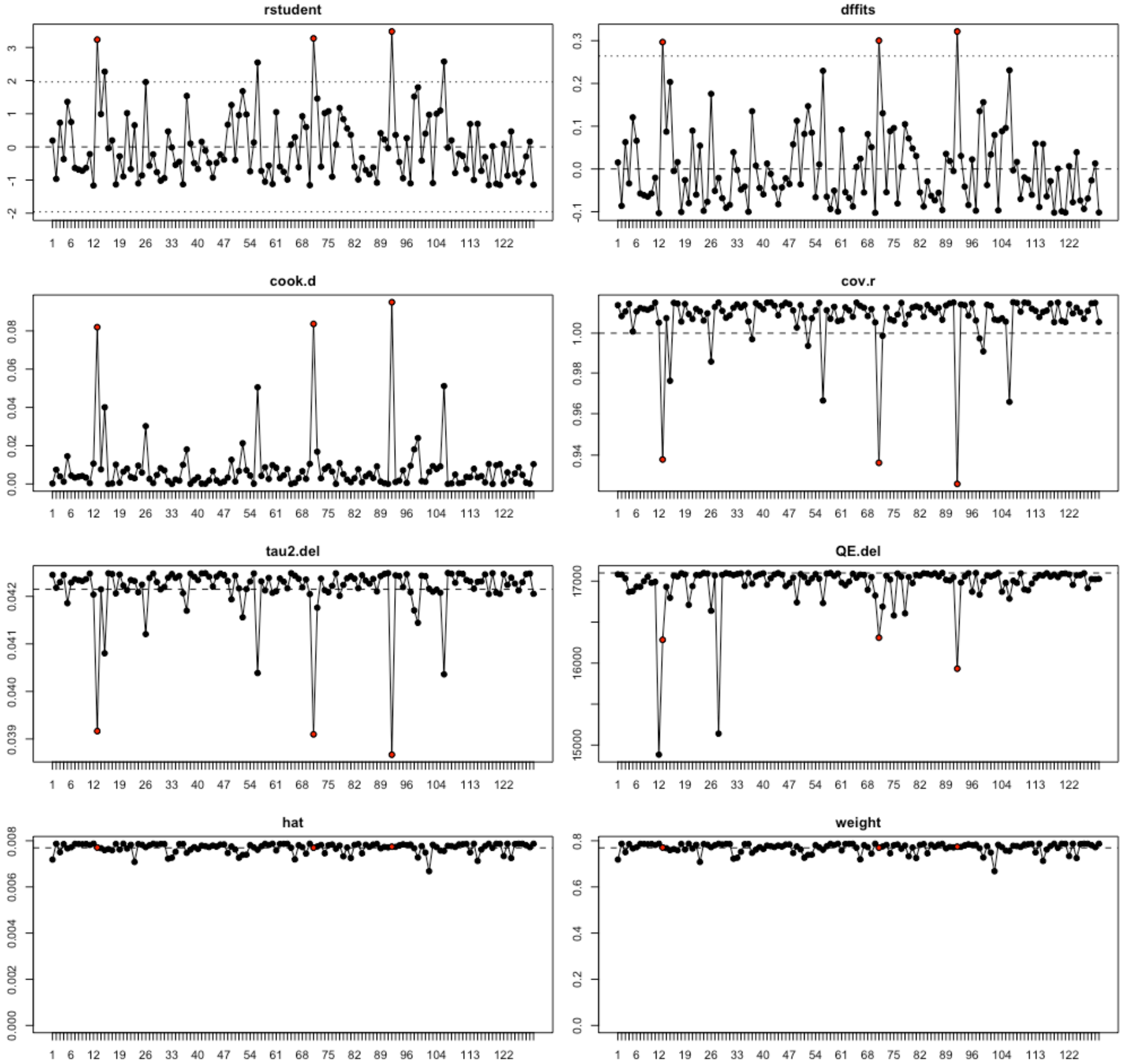

	rstudent	dffits	cook.d	cov.r	tau2.del	QE.del	hat	weight	dfbs	inf
1	0.1916	0.0153	0.0002	1.0143	0.0425	17082.9796	0.0072	0.7184	0.0153	
2	-0.9668	-0.0862	0.0074	1.0087	0.0422	17080.0319	0.0079	0.7867	-0.0862	
3	0.7276	0.0628	0.0040	1.0110	0.0423	17032.5274	0.0075	0.7506	0.0628	
4	-0.3692	-0.0339	0.0012	1.0149	0.0425	16869.8299	0.0079	0.7855	-0.0339	
5	1.3615	0.1207	0.0145	1.0008	0.0419	16878.3225	0.0077	0.7666	0.1207	
6	0.7520	0.0658	0.0043	1.0110	0.0423	16933.0023	0.0077	0.7717	0.0658	
7	-0.6369	-0.0574	0.0033	1.0128	0.0424	16932.4737	0.0079	0.7863	-0.0574	
8	-0.6828	-0.0614	0.0038	1.0124	0.0423	16999.1365	0.0079	0.7860	-0.0614	
9	-0.7205	-0.0647	0.0042	1.0119	0.0423	17056.1786	0.0078	0.7850	-0.0647	
10	-0.6338	-0.0571	0.0033	1.0129	0.0424	16979.7631	0.0079	0.7859	-0.0571	
11	-0.2177	-0.0205	0.0004	1.0156	0.0425	16991.5842	0.0078	0.7819	-0.0205	
12	-1.1645	-0.1033	0.0107	1.0053	0.0420	14882.9925	0.0079	0.7871	-0.1033	
13	3.2361	0.2967	0.0819	0.9379	0.0392	16284.1758	0.0077	0.7704	0.2967	*

14	0.9908	0.0872	0.0076	1.0077	0.0421	16930.7359	0.0077	0.7678	0.0872	
15	2.2696	0.2034	0.0401	0.9761	0.0408	16796.5796	0.0076	0.7593	0.2034	
16	-0.0415	-0.0048	0.0000	1.0155	0.0425	17065.0746	0.0076	0.7640	-0.0048	
17	0.1968	0.0161	0.0003	1.0151	0.0425	17056.8114	0.0076	0.7588	0.0161	
18	-1.1342	-0.1007	0.0101	1.0059	0.0421	17096.0449	0.0079	0.7863	-0.1007	
19	-0.2820	-0.0258	0.0007	1.0149	0.0425	17081.7015	0.0076	0.7615	-0.0258	
20	-0.8932	-0.0798	0.0064	1.0097	0.0422	16710.2394	0.0079	0.7871	-0.0798	
21	1.0190	0.0896	0.0080	1.0072	0.0421	16939.0728	0.0077	0.7662	0.0896	
22	-0.6705	-0.0602	0.0036	1.0124	0.0423	17073.6911	0.0078	0.7825	-0.0602	
23	0.6532	0.0546	0.0030	1.0112	0.0423	17072.2398	0.0071	0.7080	0.0546	
24	-1.1026	-0.0979	0.0096	1.0064	0.0421	17099.1741	0.0079	0.7857	-0.0979	
25	-0.8613	-0.0768	0.0059	1.0101	0.0422	17092.3901	0.0078	0.7827	-0.0768	
26	1.9548	0.1758	0.0302	0.9857	0.0412	16638.2128	0.0077	0.7720	0.1758	
27	-0.5674	-0.0511	0.0026	1.0134	0.0424	17070.1835	0.0078	0.7806	-0.0511	
28	-0.2233	-0.0210	0.0004	1.0156	0.0425	15139.9092	0.0079	0.7869	-0.0210	
29	-0.7641	-0.0684	0.0047	1.0114	0.0423	17076.6629	0.0078	0.7840	-0.0684	
30	-1.0205	-0.0908	0.0082	1.0078	0.0421	17098.0266	0.0079	0.7860	-0.0908	
31	-0.9417	-0.0840	0.0071	1.0090	0.0422	17089.2267	0.0079	0.7859	-0.0840	
32	0.4677	0.0391	0.0015	1.0130	0.0424	17072.9494	0.0072	0.7221	0.0391	
33	-0.0180	-0.0026	0.0000	1.0148	0.0425	17086.6324	0.0073	0.7264	-0.0026	
34	-0.5427	-0.0481	0.0023	1.0131	0.0424	17094.1019	0.0075	0.7523	-0.0481	
35	-0.4500	-0.0410	0.0017	1.0144	0.0424	16941.3413	0.0079	0.7853	-0.0410	
36	-1.1290	-0.1002	0.0100	1.0060	0.0421	17097.4774	0.0079	0.7860	-0.1002	
37	1.5385	0.1351	0.0181	0.9969	0.0417	16968.3571	0.0075	0.7478	0.1351	
38	0.1013	0.0077	0.0001	1.0154	0.0425	17059.6153	0.0076	0.7612	0.0077	
39	-0.4918	-0.0443	0.0020	1.0139	0.0424	17082.8309	0.0077	0.7723	-0.0443	
40	-0.6674	-0.0592	0.0035	1.0122	0.0423	17094.6967	0.0076	0.7642	-0.0592	
41	0.1558	0.0127	0.0002	1.0156	0.0425	16955.8189	0.0078	0.7790	0.0127	
42	-0.1154	-0.0114	0.0001	1.0157	0.0425	17034.8154	0.0078	0.7763	-0.0114	
43	-0.4822	-0.0435	0.0019	1.0140	0.0424	17081.8672	0.0077	0.7726	-0.0435	
44	-0.9277	-0.0824	0.0068	1.0092	0.0422	17097.9440	0.0078	0.7791	-0.0824	
45	-0.4780	-0.0432	0.0019	1.0140	0.0424	17078.3213	0.0077	0.7748	-0.0432	
46	-0.2326	-0.0218	0.0005	1.0155	0.0425	16937.8984	0.0078	0.7838	-0.0218	
47	-0.3835	-0.0351	0.0012	1.0148	0.0424	16977.0371	0.0078	0.7842	-0.0351	
48	0.6710	0.0576	0.0033	1.0116	0.0423	17042.5241	0.0075	0.7470	0.0576	
49	1.2659	0.1127	0.0126	1.0028	0.0419	16740.7374	0.0078	0.7754	0.1127	
50	-0.3973	-0.0358	0.0013	1.0143	0.0424	17086.2552	0.0076	0.7623	-0.0358	
51	0.9586	0.0819	0.0067	1.0077	0.0422	17048.9787	0.0073	0.7264	0.0819	
52	1.6795	0.1469	0.0213	0.9936	0.0416	16981.0270	0.0074	0.7390	0.1470	
53	0.9820	0.0848	0.0072	1.0075	0.0422	17032.1617	0.0074	0.7400	0.0848	
54	-0.7383	-0.0661	0.0044	1.0117	0.0423	17085.3695	0.0078	0.7813	-0.0661	
55	0.1343	0.0107	0.0001	1.0155	0.0425	17029.6926	0.0077	0.7712	0.0107	
56	2.5476	0.2294	0.0505	0.9664	0.0404	16731.4910	0.0076	0.7605	0.2295	
57	-0.7261	-0.0648	0.0042	1.0117	0.0423	17091.8119	0.0078	0.7761	-0.0648	
58	-1.0505	-0.0934	0.0087	1.0073	0.0421	17099.1407	0.0079	0.7866	-0.0934	
59	-0.5629	-0.0508	0.0026	1.0135	0.0424	17062.9141	0.0078	0.7818	-0.0508	
60	-1.1198	-0.0995	0.0099	1.0061	0.0421	17092.5681	0.0079	0.7869	-0.0995	
61	1.0497	0.0919	0.0084	1.0066	0.0421	16981.7212	0.0076	0.7580	0.0919	
62	-0.5986	-0.0541	0.0029	1.0132	0.0424	16949.7241	0.0079	0.7860	-0.0541	
63	-0.7505	-0.0674	0.0046	1.0116	0.0423	16993.3149	0.0079	0.7864	-0.0674	
64	-0.9864	-0.0879	0.0077	1.0083	0.0422	17089.4374	0.0079	0.7866	-0.0879	
65	0.0682	0.0048	0.0000	1.0156	0.0425	17042.8421	0.0077	0.7697	0.0048	
66	0.2937	0.0240	0.0006	1.0139	0.0424	17079.7606	0.0072	0.7193	0.0240	
67	-0.6113	-0.0550	0.0030	1.0130	0.0424	17074.6037	0.0078	0.7808	-0.0550	
68	0.9254	0.0815	0.0066	1.0087	0.0422	16893.9087	0.0077	0.7722	0.0815	
69	0.5985	0.0511	0.0026	1.0123	0.0424	17050.4357	0.0074	0.7445	0.0511	
70	-1.1546	-0.1025	0.0105	1.0055	0.0420	16824.6371	0.0079	0.7871	-0.1025	
71	3.2726	0.3001	0.0837	0.9364	0.0391	16309.7501	0.0077	0.7696	0.3001	*
72	1.4585	0.1303	0.0168	0.9987	0.0418	16688.2669	0.0078	0.7751	0.1303	
73	-0.6020	-0.0542	0.0030	1.0131	0.0424	17067.2445	0.0078	0.7820	-0.0542	
74	1.0195	0.0884	0.0078	1.0070	0.0421	17019.5249	0.0075	0.7458	0.0884	
75	1.0776	0.0958	0.0092	1.0064	0.0421	16580.3275	0.0078	0.7802	0.0958	
76	-0.9047	-0.0807	0.0065	1.0095	0.0422	17093.1340	0.0078	0.7840	-0.0807	

77	0.0728	0.0052	0.0000	1.0155	0.0425	17054.0694	0.0077	0.7654	0.0052		
78	1.1753	0.1046	0.0109	1.0046	0.0420	16606.9063	0.0078	0.7792	0.1046		
79	0.8346	0.0714	0.0051	1.0095	0.0422	17050.0356	0.0073	0.7323	0.0714		
80	0.5557	0.0482	0.0023	1.0131	0.0424	16975.7880	0.0077	0.7707	0.0482		
81	0.3656	0.0303	0.0009	1.0137	0.0424	17075.2986	0.0073	0.7251	0.0303		
82	-0.6074	-0.0547	0.0030	1.0131	0.0424	17067.4628	0.0078	0.7821	-0.0547		
83	-0.9858	-0.0878	0.0077	1.0083	0.0422	17096.2215	0.0079	0.7856	-0.0878		
84	-0.3265	-0.0293	0.0009	1.0144	0.0424	17090.1778	0.0075	0.7455	-0.0293		
85	-0.6998	-0.0628	0.0040	1.0121	0.0423	17076.9395	0.0078	0.7826	-0.0628		
86	-0.8262	-0.0734	0.0054	1.0105	0.0423	17096.5280	0.0077	0.7739	-0.0734		
87	-0.6172	-0.0556	0.0031	1.0130	0.0424	17057.1202	0.0078	0.7834	-0.0556		
88	-1.0799	-0.0960	0.0092	1.0068	0.0421	17099.6668	0.0079	0.7863	-0.0960		
89	0.4145	0.0355	0.0013	1.0141	0.0424	17016.1765	0.0077	0.7669	0.0355		
90	0.2228	0.0186	0.0003	1.0153	0.0425	17007.6851	0.0077	0.7731	0.0186		
91	-0.0427	-0.0049	0.0000	1.0157	0.0425	17048.9210	0.0077	0.7713	-0.0049		
92	3.4828	0.3215	0.0950	0.9264	0.0387	15932.3110	0.0077	0.7742	0.3215	*	
93	0.3582	0.0306	0.0009	1.0146	0.0424	16983.5089	0.0077	0.7736	0.0306		
94	-0.4553	-0.0413	0.0017	1.0143	0.0424	17058.7420	0.0078	0.7801	-0.0413		
95	-0.9465	-0.0843	0.0071	1.0089	0.0422	17095.5567	0.0078	0.7844	-0.0843		
96	0.2655	0.0225	0.0005	1.0153	0.0425	16869.6817	0.0078	0.7811	0.0225		
97	-1.1002	-0.0976	0.0095	1.0065	0.0421	17099.5218	0.0078	0.7838	-0.0976		
98	1.5189	0.1352	0.0181	0.9973	0.0417	16834.4502	0.0077	0.7677	0.1352		
99	1.7959	0.1561	0.0240	0.9908	0.0414	16996.7256	0.0073	0.7272	0.1562		
100	-0.4147	-0.0377	0.0014	1.0145	0.0424	17067.5828	0.0078	0.7770	-0.0377		
101	0.4001	0.0338	0.0012	1.0139	0.0424	17056.9108	0.0075	0.7490	0.0338		
102	0.9711	0.0796	0.0063	1.0069	0.0422	17074.6130	0.0067	0.6681	0.0796		
103	-1.0903	-0.0967	0.0093	1.0066	0.0421	17099.6626	0.0078	0.7828	-0.0967		
104	1.0014	0.0884	0.0078	1.0076	0.0421	16870.9717	0.0077	0.7726	0.0884		
105	1.0936	0.0958	0.0092	1.0059	0.0421	16979.4733	0.0076	0.7573	0.0958		
106	2.5726	0.2309	0.0512	0.9657	0.0404	16785.2950	0.0076	0.7555	0.2310		
107	-0.0235	-0.0033	0.0000	1.0158	0.0425	17004.6321	0.0078	0.7782	-0.0033		
108	0.1981	0.0164	0.0003	1.0154	0.0425	16978.7691	0.0078	0.7769	0.0164		
109	-0.7874	-0.0700	0.0049	1.0110	0.0423	17095.6275	0.0077	0.7731	-0.0700		
110	-0.2079	-0.0196	0.0004	1.0156	0.0425	16897.7871	0.0078	0.7843	-0.0196		
111	-0.2711	-0.0252	0.0006	1.0154	0.0425	16888.5522	0.0078	0.7848	-0.0252		
112	-0.6714	-0.0604	0.0037	1.0125	0.0423	16971.0071	0.0079	0.7862	-0.0604		
113	0.6910	0.0595	0.0036	1.0114	0.0423	17037.0163	0.0075	0.7496	0.0595		
114	-0.9982	-0.0889	0.0079	1.0082	0.0422	17073.8806	0.0079	0.7870	-0.0889		
115	0.6968	0.0585	0.0034	1.0108	0.0423	17068.8231	0.0071	0.7127	0.0585		
116	-0.7255	-0.0641	0.0041	1.0115	0.0423	17096.4808	0.0076	0.7618	-0.0641		
117	-0.3063	-0.0282	0.0008	1.0151	0.0425	17055.9036	0.0078	0.7770	-0.0282		
118	-1.1510	-0.1022	0.0104	1.0056	0.0421	17079.8222	0.0079	0.7869	-0.1022		
119	0.0219	0.0008	0.0000	1.0156	0.0425	17048.5057	0.0077	0.7694	0.0008		
120	-1.1125	-0.0988	0.0098	1.0063	0.0421	17089.6177	0.0079	0.7870	-0.0988		
121	-1.1474	-0.1018	0.0103	1.0056	0.0421	17093.2528	0.0079	0.7864	-0.1018		
122	0.0904	0.0067	0.0000	1.0148	0.0425	17081.3918	0.0073	0.7334	0.0067		
123	-0.8695	-0.0777	0.0061	1.0101	0.0422	16953.8595	0.0079	0.7869	-0.0778		
124	0.4673	0.0391	0.0015	1.0130	0.0424	17071.6260	0.0073	0.7250	0.0391		
125	-0.8242	-0.0737	0.0055	1.0107	0.0423	17073.3645	0.0079	0.7854	-0.0737		
126	-1.0498	-0.0934	0.0087	1.0073	0.0421	17096.3017	0.0079	0.7870	-0.0934		
127	-0.7703	-0.0691	0.0048	1.0114	0.0423	16914.4819	0.0079	0.7868	-0.0691		
128	-0.2880	-0.0266	0.0007	1.0153	0.0425	17025.5760	0.0078	0.7808	-0.0266		
129	0.1598	0.0130	0.0002	1.0155	0.0425	17023.0745	0.0077	0.7720	0.0130		
130	-1.1454	-0.1017	0.0103	1.0057	0.0421	17027.7078	0.0079	0.7871	-0.1017		

eAppendix 5.5 Moderating Effect:

MEDQUARG Score:

```
Mixed-Effects Model (k = 130; tau^2 estimator: REML)

tau^2 (estimated amount of residual heterogeneity):     0.0424 (SE = 0.0054)
tau (square root of estimated tau^2 value):             0.2059
I^2 (residual heterogeneity / unaccounted variability): 99.92%
H^2 (unaccounted variability / sampling variability):   1266.06
R^2 (amount of heterogeneity accounted for):            0.00%

Test for Residual Heterogeneity:
QE(df = 128) = 16780.3533, p-val < .0001

Test of Moderators (coefficient 2):
QM(df = 1) = 0.2543, p-val = 0.6141

Model Results:

         estimate      se    zval    pval    ci.lb   ci.ub
intrcpt    0.2131  0.0543  3.9258  <.0001   0.1067  0.3194  ***
mods       0.0044  0.0087  0.5042  0.6141  -0.0126  0.0213

---
Signif. codes:  0 ‘***’ 0.001 ‘**’ 0.01 ‘*’ 0.05 ‘.’ 0.1 ‘ ’ 1
```

Publication Year:

```
Mixed-Effects Model (k = 130; tau^2 estimator: REML)

tau^2 (estimated amount of residual heterogeneity):     0.0421 (SE = 0.0054)
tau (square root of estimated tau^2 value):             0.2051
I^2 (residual heterogeneity / unaccounted variability): 99.91%
H^2 (unaccounted variability / sampling variability):   1168.19
R^2 (amount of heterogeneity accounted for):            0.17%

Test for Residual Heterogeneity:
QE(df = 128) = 15855.2781, p-val < .0001

Test of Moderators (coefficient 2):
QM(df = 1) = 1.1654, p-val = 0.2803

Model Results:

         estimate      se     zval    pval    ci.lb    ci.ub
intrcpt    6.4536  5.7569   1.1210  0.2623  -4.8297  17.7369
mods      -0.0031  0.0029  -1.0795  0.2803  -0.0087   0.0025

---
Signif. codes:  0 ‘***’ 0.001 ‘**’ 0.01 ‘*’ 0.05 ‘.’ 0.1 ‘ ’ 1
```

Number of Samples Tested:

```
Mixed-Effects Model (k = 130; tau^2 estimator: REML)

tau^2 (estimated amount of residual heterogeneity):     0.0400 (SE = 0.0051)
tau (square root of estimated tau^2 value):             0.2000
I^2 (residual heterogeneity / unaccounted variability): 99.92%
H^2 (unaccounted variability / sampling variability):   1184.56
R^2 (amount of heterogeneity accounted for):            5.11%

Test for Residual Heterogeneity:
QE(df = 128) = 16757.6395, p-val < .0001
```

```
Test of Moderators (coefficient 2):
QM(df = 1) = 7.9126, p-val = 0.0049

Model Results:

         estimate      se     zval    pval    ci.lb    ci.ub
intrcpt    0.2601  0.0193  13.4658  <.0001   0.2222   0.2979  ***
mods      -0.0000  0.0000  -2.8129  0.0049  -0.0000  -0.0000   **

---
Signif. codes:  0 ‘***’ 0.001 ‘**’ 0.01 ‘*’ 0.05 ‘.’ 0.1 ‘ ’ 1
```

Medicine Category:

```
Mixed-Effects Model (k = 130; tau^2 estimator: REML)

tau^2 (estimated amount of residual heterogeneity):     0.0416 (SE = 0.0053)
tau (square root of estimated tau^2 value):             0.2040
I^2 (residual heterogeneity / unaccounted variability): 99.92%
H^2 (unaccounted variability / sampling variability):   1250.81
R^2 (amount of heterogeneity accounted for):            1.26%

Test for Residual Heterogeneity:
QE(df = 128) = 16957.0122, p-val < .0001

Test of Moderators (coefficient 2):
QM(df = 1) = 2.5673, p-val = 0.1091

Model Results:

         estimate      se    zval    pval    ci.lb   ci.ub
intrcpt    0.1975  0.0315  6.2756  <.0001   0.1358  0.2592  ***
mods       0.0160  0.0100  1.6023  0.1091  -0.0036  0.0355

---
Signif. codes:  0 ‘***’ 0.001 ‘**’ 0.01 ‘*’ 0.05 ‘.’ 0.1 ‘ ’ 1
```

Region:

```
Mixed-Effects Model (k = 130; tau^2 estimator: REML)

tau^2 (estimated amount of residual heterogeneity):     0.0412 (SE = 0.0053)
tau (square root of estimated tau^2 value):             0.2029
I^2 (residual heterogeneity / unaccounted variability): 99.92%
H^2 (unaccounted variability / sampling variability):   1241.32
R^2 (amount of heterogeneity accounted for):            2.37%

Test for Residual Heterogeneity:
QE(df = 128) = 16162.7948, p-val < .0001

Test of Moderators (coefficient 2):
QM(df = 1) = 4.0945, p-val = 0.0430

Model Results:

         estimate      se     zval    pval    ci.lb    ci.ub
intrcpt    0.2704  0.0239  11.3382  <.0001   0.2237   0.3172  ***
mods      -0.0362  0.0179  -2.0235  0.0430  -0.0713  -0.0011    *

---
Signif. codes:  0 ‘***’ 0.001 ‘**’ 0.01 ‘*’ 0.05 ‘.’ 0.1 ‘ ’ 1
```
